# Li@C_60_ as a multi-state molecular switch

**DOI:** 10.1038/s41467-019-10300-2

**Published:** 2019-05-23

**Authors:** Henry J. Chandler, Minas Stefanou, Eleanor E. B. Campbell, Renald Schaub

**Affiliations:** 1 EaStCHEM and School of Chemistry, University of St Andrews, North Haugh, St Andrews, KY16 9ST UK; 2 EaStCHEM and School of Chemistry, University of Edinburgh, David Brewster Road, Edinburgh, EH9 3FJ UK; 3 Division of Quantum Phases and Devices, School of Physics, Konkuk University, Seoul, 05029 Korea

**Keywords:** Carbon nanotubes and fullerenes, Molecular electronics

## Abstract

The field of molecular electronics aims at advancing the miniaturization of electronic devices, by exploiting single molecules to perform the function of individual components. A molecular switch is defined as a molecule that displays stability in two or more states (e.g. “on” and “off” involving conductance, conformation etc.) and upon application of a controlled external perturbation, electric or otherwise, undergoes a reversible change such that the molecule is altered. Previous work has shown multi-state molecular switches with up to four and six distinct states. Using low temperature scanning tunnelling microscopy and spectroscopy, we report on a multi-state single molecule switch using the endohedral fullerene Li@C_60_ that displays 14 molecular states which can be statistically accessed. We suggest a switching mechanism that relies on resonant tunnelling via the superatom molecular orbitals (SAMOs) of the fullerene cage as a means of Li activation, thereby bypassing the typical vibronic excitation of the carbon cage that is known to cause molecular decomposition.

## Abstract (web summary)

Molecular switches that can access more than just a few states are difficult to realize. Here, the authors use scanning tunnelling microscopy and spectroscopy to show that a surface-bound Li@C_60_ endofullerene can be switched between 14 molecular states—distinguished by the location of the Li atom inside the fullerene cage—by resonant tunnelling through the superatom molecular orbitals.

## Introduction

Molecular electronics was born from the desire to continue technological progression beyond the physical limits of the semiconducting materials and technology currently employed. In 1974, Aviram and Ratner designed a theoretical rectifier made from just one molecule^1^. This radical new concept has subsequently led to a vast array of novel single-molecule devices developed to mimic the electronic functionalities of their macroscopic counterparts, such as wires, and transistors^2–5^. Amongst the various nanoscale components reported, molecular switches figure predominantly, not least because of their potential to drastically increase data storage density^6–12^. Nowadays, silicon technology requires of the order of one million atoms to store one bit (i.e. 0 or 1). In contrast, a single magnetic atom was recently demonstrated to act as a reliable memory bit, capable of being toggled between two magnetic states^13^. This represents the ultimate miniaturisation of an electronic component.

One strategy currently being explored relies on the use of more complex molecules with the aim of gaining access to more states than just two (0 and 1), thereby allowing storage density to increase even further. Molecular switches with multiple states, like that observed by Auwärter et al. (switching occurs amongst four distinct molecular states) match these criteria^6^. But the need for complex molecules often results in large species with intricate chemistry. One family of molecules that has garnered considerable attention is that of the endohedral fullerenes due to their stable carbon shell that acts to shield the encapsulated species^7,14–16^. Fullerenes have the advantage that, if chosen appropriately, the internal species can potentially be manipulated without compromising molecular integrity, making such species appealing for incorporating into nano-architectures. The fullerene studied in the following work, Li@C_60_, is particularly interesting because of its near-spherical shape and the loss of symmetry due to the off-centre position of the Li. A range of values have been theoretically predicted for the off-centre displacement of Li for gas-phase molecules (1.2–1.5 Å), with an energetically favourable coordination towards a C_6_ hexagonal face^16–26^. This is supported by second harmonic generation studies on films containing Li@C_60_^21^, third-order susceptibility studies on Li@C_60_ solutions^27^, and x-ray diffraction studies on [Li@C_60_]^+^PF_6_^−^ crystals^28^. Migration of the Li between the faces of the isolated molecule has been studied theoretically^16,24^ implying that the Li can easily move between local minima at elevated temperatures. Experimental evidence for the motion of Li within the cage for temperatures above 100–150 K has been obtained in THz spectroscopy^29,30^, and time-dependent dielectric measurements on [Li@C_60_]^+^PF_6_^−^ crystals^31^.

As first predicted 25 years ago^32^, the prototypical endofullerene Li@C_60_ could hence potentially reveal multi-state switching capabilities by exploiting the relocation of the off-centred Li atom within the carbon cage. The following work demonstrates a 14-state switching behaviour of individual Li@C_60_ molecules condensed on a Au(111) surface using low temperature, ultrahigh-vacuum scanning tunnelling microscopy and spectroscopy. Furthermore, careful analysis of variations in the superatom molecular orbital energies (SAMO^33,34^) allows the potential energy landscape inside an endofullerene to be explored.

## Results

### Identification of Li@C_60_ molecules within fullerene islands

**Fig. 1:**
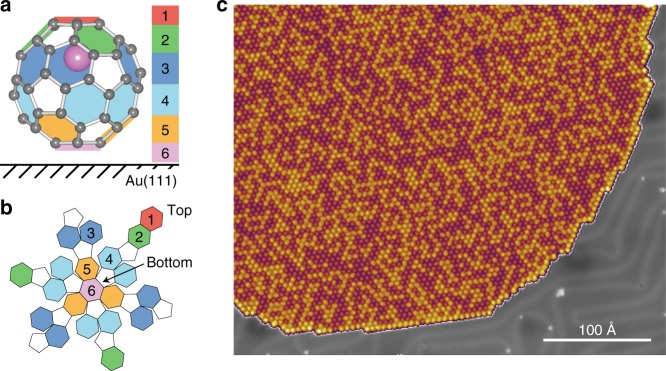
The Li@C_60_ molecule on a Au(111) surface. Ball-and-stick models of a Li@C_60_ molecule adsorbed on a surface as seen **a** from a side perspective, and **b** as a deconstructed diagram (without the Li). For a colour-coded Schlegel diagram of a C_60_ see Supplementary Note 1. The model in **a** shows the off-centre displacement of the Li atom when in level 1. **c** STM image of a large hexagonally close-packed molecular island on the Au(111) surface. Image acquired at +1.0 V, 0.1 nA

The premises of our interpretation rely on the fact that the host molecule possesses 20 C_6_ hexagonal faces, hence potentially enabling Li@C_60_ to act as a 20-state molecular switch. With all measurements being performed near liquid-helium temperatures, we rule out migration of the Li within the cage^16^ unless purposefully induced by our STM experiments. In the gas phase, the 20 Li-cage coordinations are degenerate. This will not be the case when adsorbed on a metal surface. The presence of the Au(111) support is expected to lift the degeneracy for C_6_ faces situated at different heights from the Au surface. Our recent STM studies^35^ show that the Li@C_60_ is always oriented with a C_6_ face sitting on the Au. It follows that six different Li-cage coordination Z-levels can be expected as illustrated in Fig. 1a, b (note the colour scheme used throughout this paper to identify each Z-level). Levels 1 (red) and 6 (pink) each refer to a single C_6_ face, furthest and closest to the Au(111) surface, respectively. Levels 2 (green) and 5 (orange) each refer to three C_6_ faces arranged in a three-fold symmetric distribution around the cage. These two levels are mirror-symmetric. Finally, levels 3 (blue) and 4 (cyan) are also mirror-symmetric, and refer each to six C_6_ faces arranged near the equator in three pairs of two adjacent faces (i.e. sharing one C = C double bond). Therefore, the distribution of C_6_ faces from levels 1 to 6 follows the sequence 1–3–6–6–3–1. Figure 1a illustrates in particular the Li-cage coordination for level 1, in which the Li is furthest from the metal support. Recent DFT calculations indicate that this represents the most energetically stable adsorption configuration on a Cu(111) surface^26^. Our experimental data for a Au(111) substrate are consistent with this prediction^36^.

**Fig. 2:**
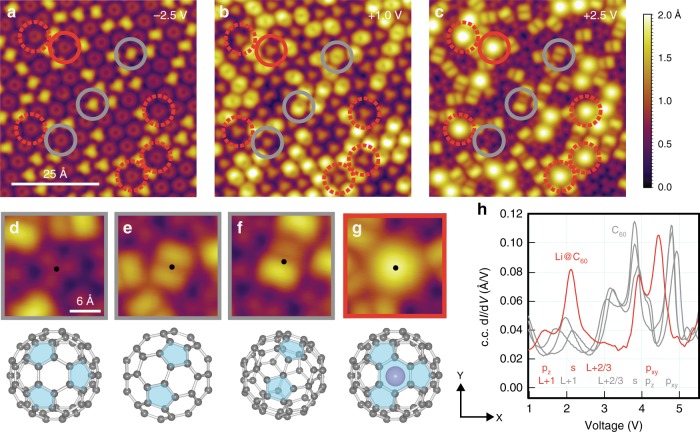
Representative STM images of C_60_/Li@C_60_ on Au(111), and C_60_ versus Li@C_60_ discrimination. **a**–**c** STM images of the same area acquired at varying tunnelling biases: **a** –2.5 V, 0.1 nA; (**b**) +1 V, 0.1 nA; (**c**) +2.5 V, 0.1 nA. **d** M-C_6_ fullerene (adsorbed through a hexagonal face). **e** C_6_–C_6_ fullerene (adsorbed through a C = C double bond joining two C_6_ faces). **f** Apex fullerene (adsorbed on a C atom adjoining two C_6_ and one C_5_ faces). **g** Li@C_60_ (always adopting the C_6_ adsorption orientation). Images **d**–**g** obtained for +2.5 V. **h** Constant-current d*I*/d*V* spectra from the four different molecular features seen in **d**–**g**

Figure 1c displays a constant-current STM image of a large island of fullerenes adsorbed on the Au(111) single crystal. The herringbone reconstruction is visible on the clean surface but is relaxed underneath the close packed island of C_60_/Li@C_60_ which adopts the well-known commensurate (2√3 × 2√3)*R*30° arrangement^37,38^. The variation in apparent height within the island, also observed by other groups for pure C_60_^36,39^, is due to different rotational orientations of C_60_, and not due to the presence of encapsulated Li atoms, as discussed by Stefanou et al. ^35^. The Li@C_60_ molecules in the midst of the island are revealed by imaging the same area with a variety of different tunnelling biases (–2.5, +1 and +2.5 V) as exhibited in Fig. 2a–c, respectively. Figure 2a, b show the different C_60_ rotational orientations previously reported in the literature^39^. However, upon imaging the same area at +2.5 V, ~13% of the fullerenes show a remarkable apparent height increase and appear circular as opposed to all other topographic signatures (Fig. 2c, dashed circles). These are the features that are attributed to Li@C_60_^26,35^. The presence of empty C_60_ on the substrate is attributed to decay processes occurring in the molecular evaporator and is consistent with the presence of C_60_ in the gas phase mass spectra obtained under similar annealing and evaporation treatment^35^.

Figure 2d–g display high magnification STM images of the four different species identified in Fig. 2c (solid circles). The corresponding ball-and-stick models illustrate the rotational alignment with respect to the support (on-top views, the blue shaded C_5_ areas indicate how the orientation is determined^40,41^, see Supplementary Note 2). Figure 2d shows a C_60_ adsorbed to the surface through a hexagonal face (C_6_). Amongst such C_6_ fullerenes, there are two possible orientations that are 180° rotations of each other. These are referred to as the majority species (labelled M-C_6_ and comprising 87% of all C_6_ fullerenes including C_60_ and Li@C_60_) and the minority species (m-C_6_, 13% of all C_6_ fullerenes). The M-C_6_ and m-C_6_ adsorption registries are rendered energetically inequivalent due to second-layer effects of the substrate. Figure 2g shows the large and bright circular shape we attribute to Li@C_60_, although the topographical data alone is an insufficient proof of Li content. For this, we compile in Fig. 2h single-point constant-current d*I*/d*V* spectroscopy data acquired on the four species. The three grey-coloured d*I*/d*V* traces show the C_60_ data to be almost independent of rotational alignment (peak assignments follow those reported in our recent PES-STM-DFT study^35^). These observations are in line with Schull and co-workers^39^. The red d*I*/d*V* trace corresponding to Li@C_60_ exhibits a completely different resonance structure, proving that this molecule is electronically inequivalent to C_60_. Careful inspection of Fig. 2a, c (dashed red circles) reveals that all cages identified with Li content share a single C_6_ adsorption orientation (with both M-C_6_ and m-C_6_ being observed, see Supplementary Note 2). This represents the most thermodynamically stable adsorption configuration, which we refer to hereafter as the native Li@C_60_ state. The most important resonance with respect to this work appears at an energy of +2.1 V, and is attributed to the Li@C_60_ S-SAMO^35^. We expect this resonance to be considerably influenced by changes in the position of the encapsulated Li within the carbon cage since the electron density associated with the SAMO orbitals penetrates the hollow core. In addition, the diffuse electron density also extends beyond the van der Waals radius of the carbon cage. This is unlike the π-symmetry LUMO orbitals which are centred on the carbon atoms, i.e. spread onto the fullerene cage^42^. The SAMO changes are then expected to have sizeable consequences on the topography, the electronic structure, and the conductance of the Li@C_60_ molecules.

### Evidence for Li@C_60_ switching

**Fig. 3:**
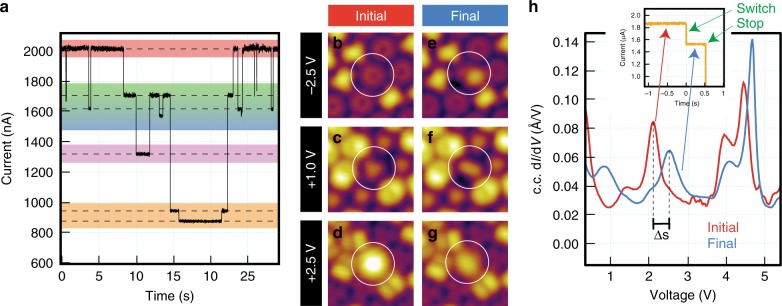
Microscopy and spectroscopy evidence for Li@C_60_ switching. **a** *I*(*t*) spectrum illustrating conductance changes of the Li@C_60_ upon the application of +5.0 V and 2.0 μA over 30 s. The reversible alterations in the conductance are seen to be confined to several distinct levels. **b**–**h** Before and after STM data illustrating a single switch event for a Li@C_60_. **b** and **e** Filled states images at –2.5 V, 0.1 nA; **c** and **f** Empty states images at +1 V, 0.1 nA; **d** and **g** Empty states images at +2.5 V, 0.1 nA, for which the contrast is dominated by the S-SAMO resonance. **h** Constant-current d*I*/d*V* spectra acquired before (red) and after (blue) the switch event reported in **b**–**g**. This direct comparison allows for spectroscopic identification of the energy shift of the S-SAMO resonance. The inset displays the *I*(*t*) data (acquired at 5 V and 1.85 μA) used to monitor the switch event by recording the associated reduction in conductance

The viability of Li@C_60_ to perform as a switch can be tested by carrying out single-point *I*(*t*) measurements^6–9,12,43^. An example is reported in Fig. 3a, acquired with +5.0 V and 2.0 μA. Over 30 s of acquisition, several abrupt conductance changes are observed. This represents a good indication of multiple and reversible switch events taking place, involving distinct molecular states (their identification is discussed in Supplementary Note 8). In order to provide further evidence for switching (an alternative approach describing a single molecule being switched sequentially is reported in Supplementary Note 3), we induce a single switch event over many molecules in their native state by injecting an identical excitation (+5.0 V and 1.95 ± 0.15 μA). Analysis of the switching behaviour is achieved by recording three pieces of information for each switch event: (1) an *I*(*t*) spectrum to identify the change in conductance involved (Supplementary Note 8), (2) STM images recorded at three biases (–2.5, +1 and +2.5 V) before and after the switch event (Supplementary Note 5), and (3) d*I*/d*V* spectra before and after (Supplementary Note 4). An example of such a procedure is reported in Fig. 3b–h.

The first three STM images (Fig. 3b–d) and the constant-current d*I*/d*V* spectrum (Fig. 3h red trace) match the data reported in Fig. 2a–c, describing a native Li@C_60_ adsorbed to the Au(111) surface in the M-C_6_ orientation prior to any induced switch. The excitation event is monitored by the conductance trace in the inset of Fig. 3h. It shows a stable molecule suddenly triggered such that the measured current reduces by ~400 nA, after which the applied excitation is removed. Post-switch, the blue d*I*/d*V* spectrum in Fig. 3h exhibits some differences in relative intensity but only small energy changes for the higher energy resonances (>3 V). However, and most importantly, a significant upwards shift of around 500 mV for the S-SAMO (to +2.6 V) is observed. Changes are also observed at lower energies (below 1.5 V) but these are difficult to interpret since the electronic features arise from a superposition of the LUMO+1 and P_z_-SAMO resonances^35^. The STM image of the filled states (–2.5 V, Fig. 3e) now shows the switched fullerene as an asymmetric kidney bean-shaped feature with two lobes of different apparent heights. The STM image at +1 V (Fig. 3f) shows an oval signature of uniform height with the same in-plane axis as the kidney bean at –2.5 V. The STM image taken at +2.5 V (Fig. 3g) appears similar to that recorded before the switch event (compare with Fig. 3d) except that the apparent height is considerably reduced.

The data presented in Fig. 3b–h can only be interpreted as a result of the inner Li atom switching its position within the fullerene cage. First, both microscopy and spectroscopy data acquired after the trigger event bear no resemblance with the corresponding data acquired on empty fullerenes. This indicates that Li ejection or even cage decomposition can be ruled out in the case discussed here (we will prove that Li ejection can occur later). Second, the 500 mV energy shift of the S-SAMO resonance demonstrates a significant change in the interaction of this orbital with the metal surface after the trigger event. This must result from Li relocation within the cage. Consequently, the reduction of apparent height seen in the STM images acquired at +2.5 V (Fig. 3d–g) is a direct result of the S-SAMO resonance shifting upwards in energy. Third, the asymmetry observed in the STM images (for instance the kidney bean shape in Fig. 3e) can only be understood by considering the likely lateral relocation of the Li atom within the cage. Finally, rotation of the entire endofullerene, rather than simply migration of the Li, can been excluded since the positions at which the switched Li is observed are discrete and coincide with the geometrical structure of the cage in the native state of the fullerene as discussed in detail below.

**Fig. 4:**
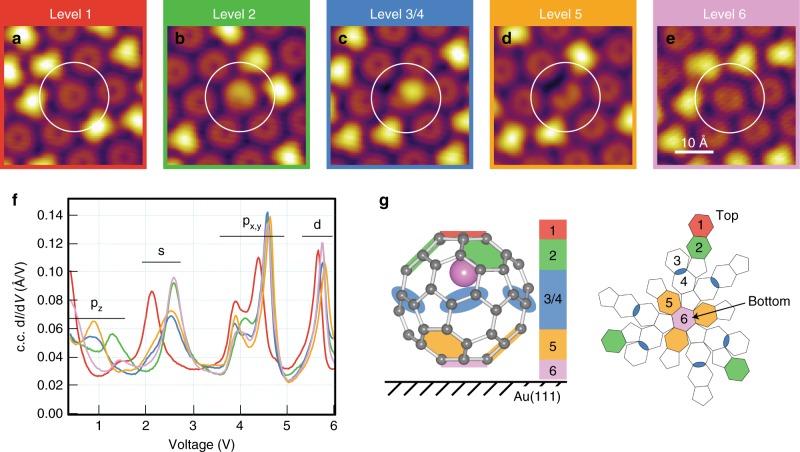
Identification of 14 switched states. All STM images acquired at –2.5 V, 0.1 nA. **a** Level 1: Li@C_60_ in the native M-C_6_ state with the Li closest to the uppermost hexagonal face. **b** Level 2: the bright protrusion can be seen to be located at three off-centre positions rotated by 120°, yielding three distinct states. **c** Levels 3 and 4 (termed level 3/4, see text): the kidney bean shape can be seen to adopt six rotational orientations, yielding six states. **d** Level 5: the dark depression can be seen to be located at three off-centre positions rotated by 120°, yielding three states. **e** Level 6: a single state similar in appearance to **a** but electronically different (see **f**). **f** Constant-current d*I*/d*V* spectra for each of the species in **a**–**e**; see also Supplementary Note 4. All data coloured according to the level they relate to. **g** *Left*: Ball-and-stick model of Li@C_60_ as seen from a side perspective, and *right***:** as a deconstructed diagram. These resemble Fig. 1a, b but are refined to highlight the actual Li coordinations experimentally identified. See also Supplementary Note 1

The measurements in Fig. 3b–h and subsequent analysis reported above were successfully repeated 270 times. Each selected endofullerene was excited only once to ensure the starting configuration of the molecule was consistent throughout all measurements, that is, the native Li@C_60_ in the C_6_ orientation. We therefore reduce the complexity of analysis to the investigation of the effects of a given electronic excitation on the switch outcome (i.e. the nature of the final state). The results are summarised in Fig. 4 and compiled in Supplementary Notes 4, 5, and 8.

### Identification of 14 distinct Li-cage coordinations

Close inspection of the STM images and the constant-current d*I*/d*V* spectra acquired before and after excitation results in the identification of 14 distinct species. Several of these exhibit identical but rotated topographic signatures, yielding the five topographic groups reported in Fig. 4a–e. The species in Fig. 4a corresponds to the native Li@C_60_ in the M-C_6_ orientation prior to any induced switch (all switch events reported hereafter derive from this initial orientation; similar results and analysis relying on the m-C_6_ initial orientation yield identical conclusions and are reported in Supplementary Note 5 for completeness). The species in Fig. 4b is characterised by a bright protrusion off-centre in the direction of a close-packed row of the molecular island. In fact, the protrusion can be observed to be off-centre in three of the possible six high-symmetry directions (see Supplementary Note 5). This indicates this species exists in three orientations rotated by 120°. Similarly, the species in Fig. 4d, which is characterised by a dark depression off-centre in a high-symmetry direction of the molecular overlayer, exists also in three possible orientations rotated by 120°. However, these are different from those adopted by the species in Fig. 4b, indicating an apparent rotation of 180° between the two species. Figure 4c shows the coordination already described in Fig. 3e and referred to as the kidney bean species. The associated bright protrusion is also observed to be off-centre, but in this case pointing in a direction bisecting two adjacent high-symmetry directions of the molecular island. There are six equivalent bisecting directions, and each of these is seen to be adopted by the kidney bean species. Hence this species exists in six orientations rotated by 60°. Finally, Fig. 4e shows a topography very similar to Fig. 4a, but its electronic structure is very different, as seen from the constant-current d*I*/d*V* spectra reported in Fig. 4f.

The number of orientations each topographic group adopts in the sequence of the STM images shown in Fig. 4 from (a) to (e), and in Supplementary Note 5, is 1–3–6–3–1. This is to be compared to the 1–3–6–6–3–1 sequence describing the number of C_6_ hexagonal faces a fullerene cage has. The natural conclusion that ensues is that the STM images must reflect various Li-cage coordinations. Proper comparison of the orientation sequence measured and expected for a native M-C_6_ registry allows us to identify Fig. 4a, b, d, e with Z-levels 1, 2, 5 and 6 respectively. We note that this validates the recent DFT-based prediction that Li@C_60_ natively adsorbs with the Li atom furthest from the metal surface^26^. However, the identification of levels 3 and/or 4 with Fig. 4c is less trivial. These levels are each made of six Li-cage coordinations. Yet, only one species is observed to possess six orientations which do not coincide with those of either level, but rather fall exactly in-between the two expectations. We hence come to three possible conclusions with regards to Fig. 4c. (i) Only one of either level 3 or 4 is realised, whilst the other is not accessible and hence not observed. We fail to rationalise a credible limiting argument. (ii) Both levels 3 and 4 are realised but their STM signatures are indistinguishable. We dismiss this argument since levels 3 and 4 discernibly differ by 15°. (iii) Our preferred conclusion: intermolecular interactions arising from the close-packed neighbouring cages are sufficient to alter the potential energy landscape of the Li atom within the carbon cage, so that the 12 near-equatorial C_6_ locations of levels 3 and 4 are merged into six perfectly equatorial locations corresponding to the C = C double bond adjoining two C_6_ faces, one of level 3 and one of level 4, as shown in Fig. 4g (compare with Fig. 1a, b). This interpretation not only explains that six Li-cage coordinations are observed rather than the expected 6–6, it also rationalises the rotational alignment observed, and it explains the asymmetry associated with the kidney bean shape (as justified in Supplementary Note 7). We refer henceforth to these unexpected six Li-cage coordinations as level 3/4.

A compelling argument for the involvement of level 3/4 stems from statistical analysis of final state frequencies. Starting from the native Li-cage coordination of level 1, the measured probabilities to switch to levels 2, 3/4, 5, and 6 are 20%, 52%, 26%, and 2%, respectively. These probabilities do not correlate with the Li–Au(111) separation distance. Rather, they indicate that the measured 3–6–3–1 = 13 Li-cage coordinations (14 minus the native one) are equally probable after excitation, for which we expect 23 ± 3%, 46 ± 3%, 23 ± 3%, and 8 ± 2%, respectively (errors are standard deviations associated with 270 random draws of four outcomes with weighted probabilities of 3–6–3–1).

As mentioned earlier, since the SAMO resonances are cage-centred (see refs ^35,42^ and Supplementary Note 4), we anticipate the relocation of the encapsulated Li atom to influence the energy of the resonances by dictating their degree of hybridisation with the metal support. Looking closely at Fig. 4f reveals indeed that the switching process shifts the energy of the S-SAMO upwards by ~500 mV, regardless of the final position of the Li. Based on this, one can hypothesise that the coupling of the diffuse S-SAMO with the *d*-states of the metal substrate plays a major role in determining the energetically preferred adsorption coordination of Li@C_60_ on the Au(111) substrate (native state identified as level 1 with the Li located furthest from the surface). The P_z_-SAMO resonance also shows a significant change but it is difficult to quantify due to its overlap with the LUMO+1. The P_x,y_-SAMO and D-SAMO resonances, all located above 3 eV, shift in energy only marginally with Li position, indicating a significantly weaker coupling to the metal substrate. Supplementary Note 4 discusses the observed SAMO energy shifts as a function of Li location within the cage in more detail.

### The switching mechanism

All Li@C_60_ switching measurements presented so far derive from tunnelling excitations of +5.0 V and 2.0 μA. These severe activation conditions raise the question of which switching mechanism is at play. Our observations can be summarised as follows: (1) only positive polarity (tunnelling into empty electronic states of Li@C_60_/Au(111)) leads to switching. At negative polarity, increasing the current and/or the bias leads to the collapse of the tunnelling junction (tip crash and/or molecule decomposition) before any switching event can be recorded. (2) At positive polarity, the switch rate increases both with current and/or voltage. 2.0 μA of current are needed at 5.0 V to observe switch rates of about 1 Hz. Further increasing the current or the voltage leads to increased rate but also more frequent tunnel junction collapse and/or molecular decomposition (as expected from the work of Schulze et al. ^44,45^). Further decreasing the current or the voltage leads to the switch rate to decrease. The lowest energy at which we have observed switching is +3.0 V, with very low rates and with a current in excess of 3 μA. At energies below 3.0 V, increasing the current leads to cage decomposition with no prior observable switching.

The two most commonly invoked non-thermal activation mechanisms in single-molecule systems are inelastic electron tunnelling (IET), and electric field (EF)^6–9,12,43^. The fact that the polarity plays such a major role suggests an EF activation. However, we rule out this mechanism on the basis of Fig. 3a whereby switching amongst different conductance states (or Li levels) is observed to be reversible. A direct IET mechanism, in which an electron activates Li diffusion within the carbon cage, can also be ruled out since this mechanism should be polarity independent. We note here that such a mechanism would otherwise be plausible following the work of the Petek group, who have beautifully demonstrated with STM that the rotational activation of Sc_3_N molecules in C_80_ cages on the Cu(110)–O(2 × 1) surface is feasible with energies as low as about ±80 meV, corresponding to the antisymmetric stretch vibration of the Sc_3_N molecule^2^. If so, we would anticipate an activation of 40–110 meV in line with the theoretically predicted energy barriers involved in the diffusion of the Li atom within a C_60_ cage^16,25^. Our experimental conditions far exceed these energies and are polarity dependent.

Schulze and co-workers have studied in detail the electron-mediated heating, heat dissipation and decomposition of C_60_ molecules on various metal surfaces, including Au(111), utilising an STM setup^44,45^. They demonstrate that resonant tunnelling through molecular states of an individual C_60_ can generate the necessary vibrational excitation (temperature) to decompose the molecule (via inelastic electron scattering with molecular vibrations). The authors limit their experimental data to excitations below 3.25 eV, i.e. encompassing only the LUMO, LUMO+1, and LUMO+2/3 resonances of C_60_, but not the SAMO resonances (the onset of the first S-SAMO resonance for C_60_ is 3.5 eV as shown in Fig. 2h). Whilst we have not undertaken a similarly comprehensive electron-induced heating study on our Li@C_60_/Au(111) system, it is reasonable to assume that the decomposition behaviour at lower energies is similar to that of C_60_/Au(111) with the exception that the tunnelling currents required for decomposition are overall larger for Li@C_60_ due to a stronger coupling with the substrate^35,45^. Molecular heating of the carbon cage, and ultimately decomposition, is achieved by resonant tunnelling mainly through the LUMO+X resonances below 3 V, which exhibit an efficient coupling to the molecular vibrational modes. The LUMO+X resonances differ from the SAMO resonances in that these are carbon-atom-centred orbitals, whereas the latter are Rydberg-like, diffuse orbitals centred on the fullerene cage. Whilst the former have their density spread over the cage (hence the efficient coupling to C_60_ vibrations), the latter have their density extending beyond as well as within the cage (see e.g. Fig. 2 of ref. ^42^ for a visual representation). Resonant tunnelling through LUMOs or SAMOs is therefore expected to lead to different mechanisms and rates of molecular excitation, with the LUMOs likely to be more conducive to cage heating than the SAMOs. In particular for Li@C_60_, it is conceivable that the SAMOs facilitate access to the inner Li atom since their density also penetrates the fullerene cage. We hence propose a mechanism whereby resonant tunnelling through SAMOs is key to Li activation. The P_z_-SAMO and S-SAMO of Li@C_60_, both located below 3 V, exhibit a strong coupling to the empty states of Au(111) (see Supplementary Note 4). Resonant tunnelling through these orbitals results in an efficient hot electron removal into the metal. This represents the first experimental confirmation of the recent theoretical prediction that hot electron cooling from the S-SAMO in a supported C_60_ system is significantly less efficient than interfacial charge transfer^46^. However, the P_x,y_-SAMOs located above 3 eV, in line with our observed onset for Li activation, are significantly decoupled from the Au(111) support (see Supplementary Note 4), thus exhibiting a less efficient electron transfer into the metal. A consequence of this is a higher probability for Li activation within the carbon cage due to coupling between the P_x,y_-SAMO and the vibrational modes of the Li. The participation of the P_x,y_-SAMO orbitals in delivering electron-induced excitations can also be indirectly inferred from observations that neighbouring empty C_60_ cages are rotationally activated at distances as far as 5 nm away from a target Li@C_60_ molecule. This may be associated with efficient electron transport through diffuse delocalised bands produced via hybridisation of the quasi-degenerate P_x,y_-SAMOs of the Li@C_60_ and the neighbouring C_60_ (see Supplementary Note 6 and refs ^42,46^).

### Li atom ejection

**Fig. 5:**
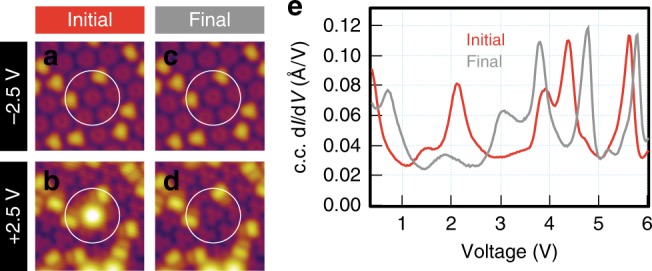
Evidence of Li atom ejection. **a**–**d** STM images before and after a Li ejection event, with **a** and **b** bearing the hallmarks of a Li@C_60_ molecule, whereas **c** and **d** correspond to an empty C_60_ molecule. Besides the Li ejection from the fullerene marked with a circle, one can observe the induced rotation of a neighbouring molecule. **e** Constant-current d*I*/d*V* spectra of the target molecule before and after Li ejection

A non-negligible fraction of excitation attempts at 5 V and ~2 μA (10%, and increasing if the voltage or current is further increased) lead to irreversible damage to the target molecule and its surroundings. Such events were discarded from the analysis reported in this paper, but their occurrence is not surprising following the work of Schulze et al. ^44,45^. More interestingly, in a handful of excitation events (<1%), we were able to witness irreversible changes to Li@C_60_ that can only be ascribed to the ejection of the Li atom without any subsequent damage to the carbon cage. The STM images in Fig. 5 were recorded on the same molecule before and after excitation. The disappearance of the bright circular protrusion characteristic of the presence of the Li within the cage in the sequential STM images acquired at +2.5 V (Fig. 5a, c) is a clear indication of Li ejection. Also, the STM sequence acquired at –2.5 V (Fig. 5b, d) shows that the ejection process does not result in visible damage to the host cage (resulting in a C_60_ M-C_6_ final state). The most compelling evidence for Li ejection stems from the constant-current d*I*/d*V* spectra recorded before and after excitation, as shown in Fig. 5e. Comparison of the red (initial) and grey (final) spectra with those reported in Fig. 2h indicate that these were acquired over a Li@C_60_ molecule and an empty C_60_ molecule, respectively. The conductance change accompanying the Li ejection was also recorded in *I*(*t*) data. Two such spectra are reported in Supplementary Note 8. It can be observed that the conductivity drops significantly with the loss of the Li, more so than as a consequence of a Li-cage coordination switch. We can presume that the loss of Li occurs through the upper C_6_ hexagon and requires at least 5 eV to succeed. This is consistent with the energetic threshold for implanting Li^+^ into a C_60_ cage^47,48^ and is also supported by ab initio molecular dynamics simulations^49^. The Li is likely ejected to the gas phase (as a consequence of the strong positive EF applied by the STM tip), since our STM images do not reveal tell-tale signs of the Li fate, e.g. the formation of an exofullerene.

### Conclusion

To conclude, we have shown through a combination of scanning tunnelling microscopy and spectroscopy that it is possible to purposefully induce reversible Li-cage coordination changes in Li endofullerenes condensed in a close-packed island of C_60_/Li@C_60_ on a Au(111) surface. By applying EFs in excess of 3 V and very high current densities (in the μA range) to selected Li@C_60_ molecules, 14 distinct Li-cage coordinations are statistically populated and unambiguously identified. To the best of our knowledge, this represents by far the largest number of accessible, reversible, and stable states observed for a surface-bound single molecule switch. However, we recognise the current lack of final-state control compared to the degree of hierarchical control at low bias voltage previously reported for the rotation of Sc_3_N inside C_80_^7^. We propose that Li activation is a result of resonant tunnelling via SAMO resonances, as initially suggested in a theoretical study by Jorn et al. ^22^. Further exploration of the isomerisation mechanism could be applied to isolated Li@C_60_ molecules. We suspect, however, that if the manipulation parameters did not merely result in molecular diffusion, we could expect 20 distinct cage coordinations due to the lack of neighbouring fullerenes affecting the internal surface potential of the Li@C_60_.

## Methods

### Li@C_60_ conditioning and deposition

Commercial, purified [Li@C_60_]^+^(PF_6_)^–^ salt (>80% purity, Idea International Inc.) was placed in a molecular evaporator consisting of a small quartz-glass capillary tube containing the molecular mixture and surrounded by a heating element. The material was heated at a range of temperatures up to 590 K for >100 h to remove the ligands and any lingering impurities. In this conditioning phase prior to deposition, some Li content is lost through a slow thermal decay process^26,35^. The temperature was then raised to 665 K for molecular sublimation, and the Au(111) surface (prepared by Ar^+^ sputtering and annealing cycles) was held at about 80 K and exposed to the effusive molecular beam for 4 min at a partial pressure of 5 × 10^–8^ mbar. The sample then underwent a post-adsorption anneal to 570 K for 30 s in order to desorb additional unwanted pollutants and induce molecular diffusion to form large, hexagonally close-packed islands of molecules. The sublimation temperature used here is compatible with what is reported for the sublimation of unfilled C_60_ molecules^36^ and was also the temperature used to prepare gas-phase targets of Li@C_60_ from the same starting material^35^.

### Scanning probe microscopy and spectroscopy

All measurements were conducted with a commercially available CreaTec ultrahigh vacuum low-temperature scanning tunnelling microscope (UHV LT-STM) using an electrochemically etched tungsten tip under ultra-high vacuum (<1 × 10^−11^ mbar) at sample temperatures of ~5 K. Constant-current STM images and constant-current d*I*/d*V* spectra (measured by lock-in technique with a superimposed modulation amplitude of 50 mV peak-to-peak and frequency of 1470 Hz) are used to identify the fullerenes.

## Supplementary information

Supplementary Information

Peer Review

## Data Availability

The microscopy data, spectroscopy data, and theoretical calculations that support the findings of this study are available in the St. Andrews Research Portal with the identifier 10.17630/d440ef8d-6ccf-4fc4-aa37-0c601eb165a3^50^.
