# Supplementary material for: Li@C60 as a multi-state molecular switch
Source: Nat Commun. 2019 May 23;10:2283. doi: 10.1038/s41467-019-10300-2 (PMC6533348; doi:10.1038/s41467-019-10300-2)
Supplement: Supplementary file 1 — Supplementary Information [file 41467_2019_10300_MOESM1_ESM.pdf]

Supplementary Information

Li@C_{60} as a multi-state molecular switch

Henry J. Chandler[1], Minas Stefanou[2], Eleanor E. B. Campbell[2,3], Renald Schaub[1]*

1. EaStCHEM and School of Chemistry, University of St Andrews, North Haugh, St Andrews, KY16 9ST, UK
2. EaStCHEM and School of Chemistry, University of Edinburgh, David Brewster Road, Edinburgh EH9 3FJ, UK
3. Division of Quantum Phases and Devices, School of Physics, Konkuk University, Seoul 05029, Korea

*Corresponding author: Renald Schaub (renald.schaub@st-andrews.ac.uk)

**The research data supporting the findings of this study are available in the St Andrews Research Portal with the identifier doi:10.17630/d440ef8d-6ccf-4fc4-aa37-0c601eb165a3.

Supplementary Note 1. Colour-coded diagrams to identify the coordination sites of Li within Li@C_{60}.

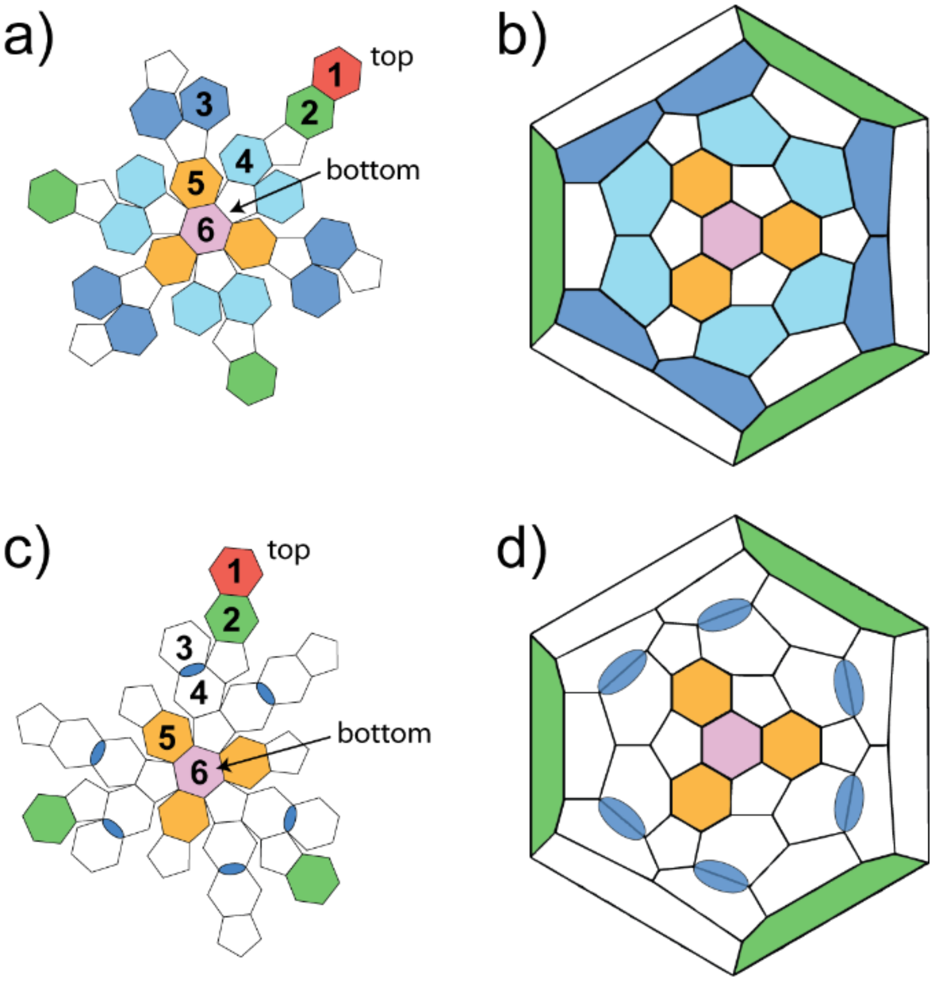

Supplementary Figure 1. Different views of the colour-coded C_{60} cage. (a) and **(c)** Deconstructed diagrams, also known as net diagrams. These two diagrams are used throughout our manuscript. However, some in the field of endohedral fullerenes are more familiar with Schlegel diagrams, as shown in **(b)** and **(d)**, when referring to the differing facets of the cage. As such, we provide here both representations for ease of comparison. Diagrams identifying the 20 different hexagonal faces available on the C_{60} illustrated by (a) a deconstructed (net) diagram and (b) a Schlegel diagram viewed through the hexagon in Level 1 (as if viewing the fullerene orthogonal to the surface). The updated understanding of the various Li coordinations is illustrated in (c) as a net diagram and (d) as a Schlegel diagram. Both distinctly highlight the coalescing of levels 3 and 4 into the equatorial species referred to as Level 3/4.

Supplementary Note 2. Population distributions and rotational coordinations for C_{60} and Li@C_{60} adsorbed on Au(111).

The following section is identical to our combined PES-STM work reported in [**1**], section 2 of its Supplementary Material. We report the same section here for completeness. The population distributions of C_{60} and Li@C_{60} and their rotational coordinations to Au(111) are reported in **Supplementary Figure 2**. The data is based on statistical analysis of many STM images acquired at different voltages. The Li@C_{60} are differentiated from C_{60} thanks to the bright circular feature appearing at +2.5 V. Statistical analysis reveals that 13% of all adsorbed fullerenes are Li@C_{60}. The rotational coordinations of the fullerenes with respect to the Au(111) surface are deduced from the shape and symmetry of features observed in the STM images at all tunnelling voltages, as reported by Gardener and co-workers [**2**]. The data acquired at –2.5 V allow identification of the fullerene faces (C_6, C_6-C_6, and C-apex) in contact with the support [**3, 4**], and hence those furthest from the support (indicated by blue shadings in the ball-and-stick models). The data acquired at +1.0 V indicate that the C_6 species (both empty and Li-filled) adsorb in two orientations rotated by 180° (as seen from the triangular or trefoil shapes), one of them being predominant (roughly 90% versus 10%), as a result of second-layer effects of the gold substrate. We refer to these mirror-symmetric orientations as majority (M-C_6) and minority (m-C_6) species. Most importantly, whilst the C_{60} molecules are seen to adsorb in three possible coordinations (C_6, C_6-C_6, and C-apex, with the C_6-C_5 and C_5 coordinations never observed on Au(111), contrary to e.g. Cu(100) [**5**]), Li@C_{60} is only observed in the C_6 coordination after sample preparation. We refer to this unique Li@C_{60} coordination as native.

	Species	Model	STM Data: –2.5 V / +1.0 V / +2.5 V	% Type	% Total
Li@C_{60}	M-C_6	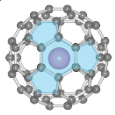	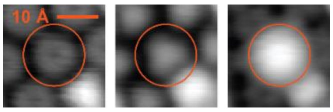	91.3 %	11.5 %
	m-C_6	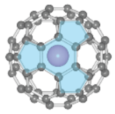	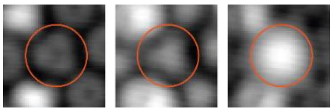	8.7 %	1.1 %
C_{60}	M-C_6	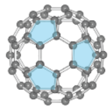	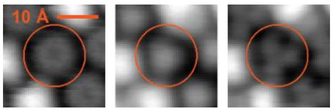	86.1 %	36.6 %
	m-C_6	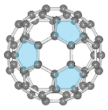	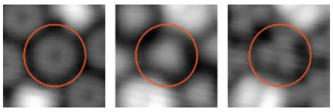	13.9 %	5.9 %
	C_6-C_6	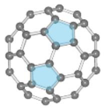	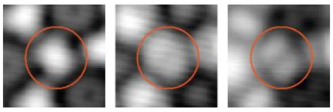		21 %
	C-apex	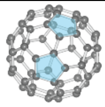	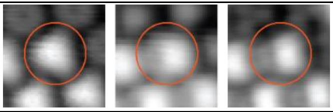		23.8 %
	C_6-C_5 & C_5	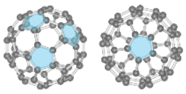	Not observed		0 %

Supplementary Figure 2. Li@C_{60} and C_{60} species identified on the Au(111) surface. Ball-and-stick models, STM images (acquired over the same area at –2.5 V, +1.0 V and +2.5 V from left to right), population ratios.

Supplementary Note 3. Experimental proof of Li@C_{60} switching.

The difficulty of applying the well documented practice to provide evidence for switching:

In order to provide evidence for switching and to elucidate the operating mechanism, a well-documented practice involves statistical analysis of many thousands of switch events measured from *I(t)* spectra acquired at different tunnelling currents and voltages [**6–12**]. This approach is simply not applicable to the present system for several reasons: (1) The conditions required to instigate reliable switching within a workable timeframe as shown in **Figure 3(a)** (+5.0 V and 2.0 µA) are very severe compared to other molecular switches reported in the literature (typically on the order of magnitude of 0.5 V and 1 nA). Our conditions for molecular excitation lie very close to the maximum the molecule/surface system is capable of accepting (beyond those conditions, damage to the molecules often results) [**13, 14**]. (2) At the very best, our switch rate can reach about 1 Hz (as shown in **Figure 3(a)**), whilst rates of up to 10-1000 Hz are easily demonstrated on other molecular switches at standard excitation parameters. (3) Most reported switches operate on two distinguishable states, with 4 [**6**] and even 6 states [**7**] representing the highest reported so far (not involving translations or rotations of the molecular switch). The Li@C_{60} molecule can potentially accommodate 20 distinguishable switch states, further complicating the analysis of the system. These technical and inherent challenges call for a different approach, hence the one we describe in our main text.

Sequential switching of a single Li@C_{60} molecule – A reversible process:

Supplementary Figures 3(a)-(k) display a series of sequential STM images acquired on a single Li@C_{60} molecule. *I(t)* spectra were recorded whilst the excitation was delivered by the STM tip to the molecule. Once a switch event was recorded by observing a change in the conductance, i.e. a step-like change in the *I(t)* data, the excitation was halted and the molecule imaged again. A constant-current *dI/dV* spectrum is also acquired over the target molecule after each excitation (**Supplementary Figure 3(l)**). Following the direction indicated by the orange arrow, the sequence of Li-coordinations achieved, and denoted by their Z-level, is: 1–2–1–3/4–1–2–1–5–1. Note that in three instances along the excitation sequence, neighbouring empty C_{60} molecules are rotated (see related discussion in **Supplementary Note 6**). Most importantly, **Supplementary Figure 3** indicates that the switching process of a single Li@C_{60} molecule is reversible. The data also shows a clear preference for the native C_6 orientation of Level 1. This is to be expected since sample preparation leads to all endofullerenes thermally stabilising into exclusively the M-C_6 or m-C_6 coordinations of Level 1. Note that the reversibility of the switch process and the increased stability of Level 1 (exhibiting highest conductance) are also observed in the *I(t)* spectrum displayed in **Figure 3(a)**.

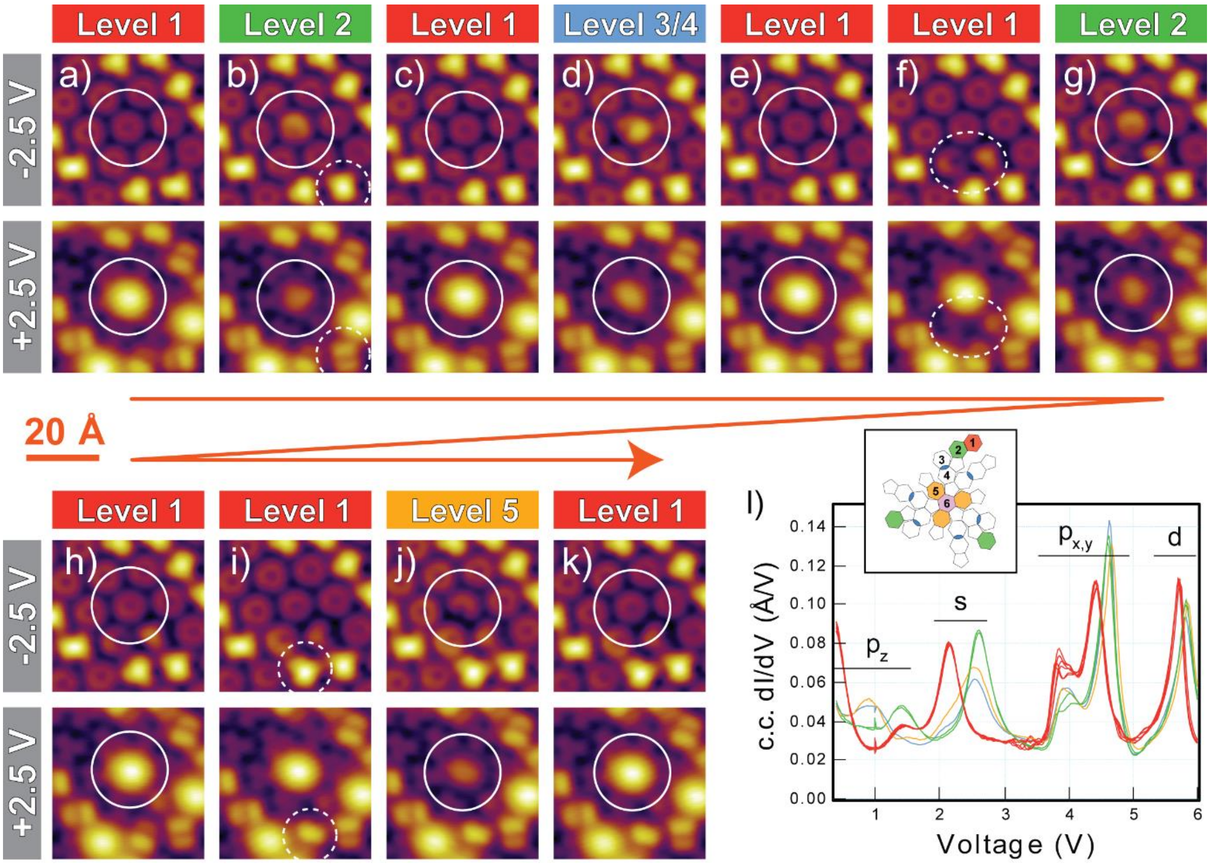

Supplementary Figure 3. Sequential switch events. (a)-(k) STM images monitoring a sequence of Li-coordination changes. The corresponding Z-levels are indicated above the STM images and coloured according to the scheme depicted in the inset of (l). The solid circles highlight the Li@C_{60} molecule, whereas the dashed ovals highlight neighbouring empty C_{60} molecules that undergo cage rotations during excitation with the STM tip held over the Li@C_{60}. The conditions used for excitation are, as reported in the main manuscript, +5 V and approximately 2 μA. **(l)** Constant-current *dI/dV* spectra acquired over each of the species imaged in (a) to (k). Inset: Ball-and-stick model illustrating the colour scheme used to identify the different Z-levels.

Supplementary Note 4. The influence of the Li position on the SAMO resonances of Li@C_{60}.

Energy shifts of the SAMO resonances of Li@C_{60} as function of Li position:

For each switching event recorded, constant-current d*I*/d*V* spectra were acquired before and after the excitation. Representative spectra are displayed in **Supplementary Figure 4**. The red traces correspond to the Li@C_{60} molecules in their native Level 1 state, that is, before excitation. One can see that the SAMO resonances are very well reproduced. All SAMO resonances, with the exception of the P_z-SAMO, are observed to shift towards higher energies once the Li position within the C_{60} cage is altered. This blue shift is more pronounced for the S-SAMO than it is for the $P_{x,y}$- and D-SAMOs. The energy changes related to the P_z-SAMO are more difficult to interpret due to the overlap with the LUMO+1. The observed shifts can be rationalised by considering the spatial extension and orientation of the SAMOs upon alteration of the Li atom position, as discussed below.

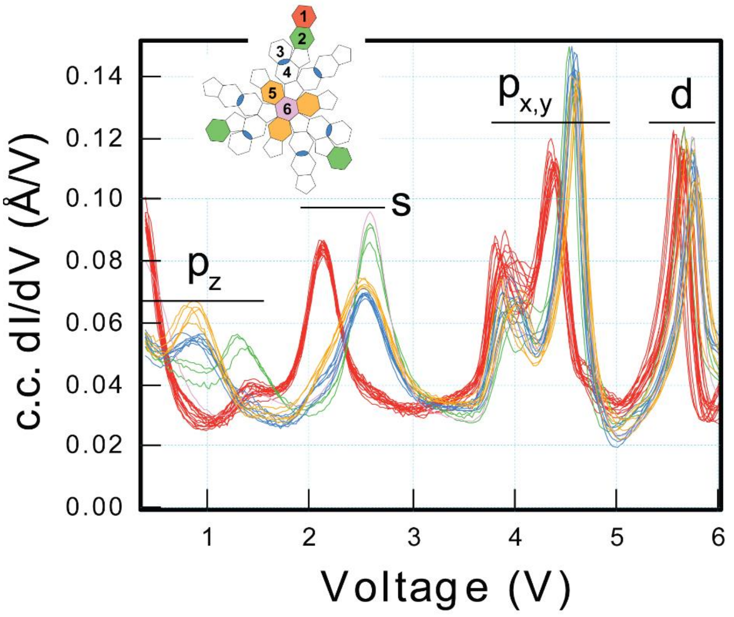

Supplementary Figure 4. Representative examples of constant-current d*I*/d*V* spectra acquired on Li@C_{60} before (red traces for native Z-level 1) and after (coloured traces for Z-levels 2, 3/4, 5, and 6) switching events. The SAMO resonances are specified.

Spatial extension and orientation of the SAMO resonances of Li@C_{60}:

A ground state DFT geometry optimisation using Gaussian 09 was performed on gas-phase Li@C_{60} using the PBE functional and the 6-31+G* basis set. In the optimised structure, the Li is displaced from the centre of the cage by 1.6 Å, towards the centre of a hexagonal ring. This value coincides well with the previously published experiments and calculations [**15–19**]. From these calculations, the atomic orbitals of the SAMOs are represented in **Supplementary Figure 5** with an iso-value of 0.009 $|e|/a_o^3$. No metal support was considered in the calculation. The indicated Au(111) support is only manually added to the figure as a reference. The distance shown between the metal support and the closest hexagonal ring was chosen as a value found in the literature for C_{60} molecules adsorbed on a Au(111) surface [**20**].

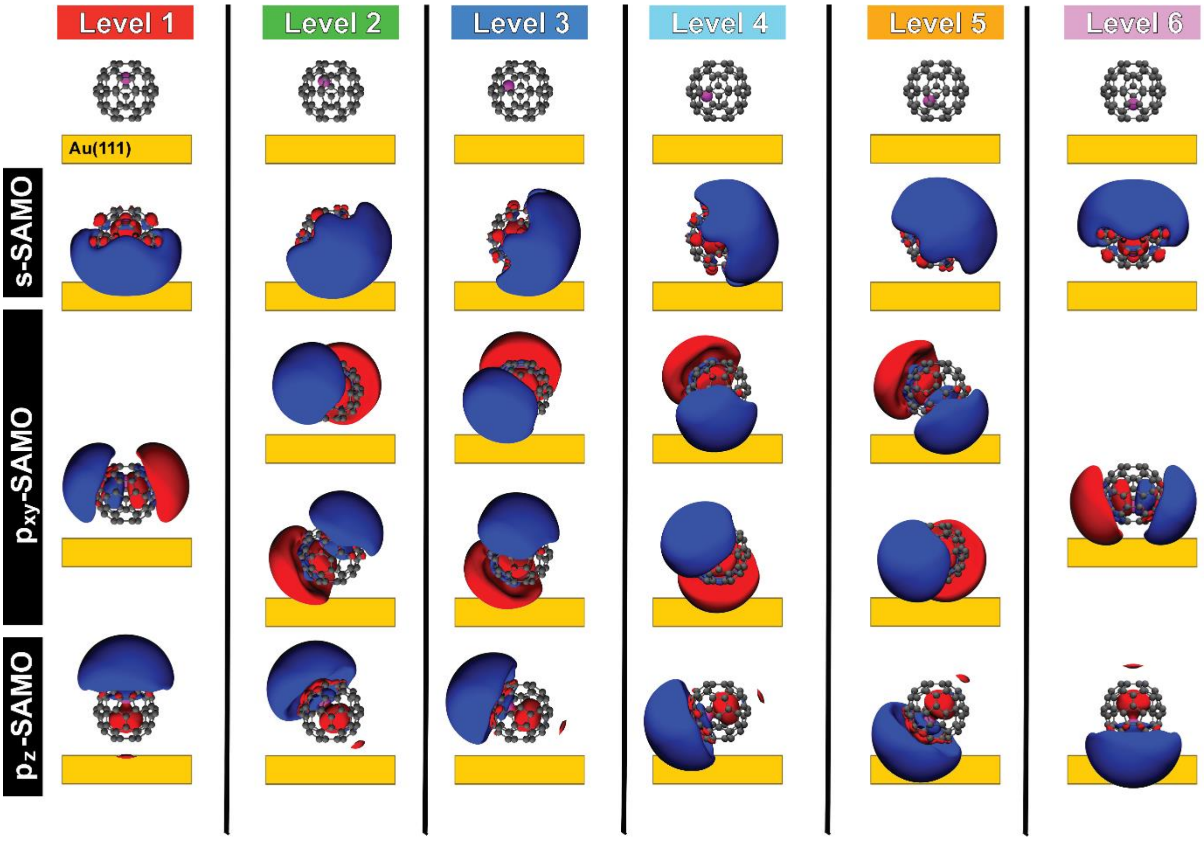

Supplementary Figure 5. Electron densities from DFT calculations for Li@C_{60} SAMOs. The data shows electron densities (iso-value of 0.009 $|e|/a_o^3$) for the S-, $P_{x,y}$- and P_z-SAMOs for each of the possible Z-levels for Li-cage coordination. The calculations are gas-phase, with no consideration of the metal support, which is manually added to the figure just for reference. This image was made with VMD. VMD is developed with NIH support by the Theoretical and Computational Biophysics group at the Beckman Institute, University of Illinois at Urbana-Champaign.

Rationalising the SAMO energy shifts observed:

Given that a significant portion of the electron density of the SAMOs is situated inside the cage and the SAMOs are distorted by the presence of the endohedral Li [**1**], the position of the Li influences the position of the SAMOs with respect to the metal support. In turn, this dictates the amount of overlap between the molecular resonances and the d-states of the metal. The degree of overlap thus governs the extent of stabilisation accompanied with energy shifts of the molecular resonances. This line of thought is strongly supported by the observation that all SAMO resonances of the Li@C_{60} are shifted downwards when compared to those of the empty C_{60} molecule, see reference [**1**] for more details on the C_{60} and Li@C_{60} comparison. **Supplementary Figure 5** shows that the P_z- and S-SAMO orbitals are pointing in opposite directions. In **Figure 4(f)**, the S-SAMO of level 1 is red shifted compared to the other levels. As can be seen from the above schematics, the S-SAMO-metal support overlap is at a maximum for that level. We therefore conclude that a larger SAMO-metal support overlap will stabilise the resonances and thus reduce their energy. This argument also explains very well why the $P_{x,y}$-SAMOs show little to no change in energy as a function of Li position within the cage, since these orbitals exhibit very little coupling with the metal substrate (their densities mostly lie in the plane above and parallel to the surface).

Supplementary Note 5. STM imaging of all 14 Li-cage coordinations identified for Li@C_{60}.

The large **Supplementary Figure 6** below reports the complete collection of STM images identifying the 14 Li-cage coordinations for both M-C_6 and m-C_6 Li@C_{60} molecules. The large data set is grouped and coloured by Z-level. Each switch event is reported with initial and final states for direct comparison. Note that all initial states correspond to the native Z-level 1. It follows that all switch events involve Li-cage coordination changes from level 1 to levels 2, 3/4, 5 or 6. Ball-and-stick models accompany every switch event to illustrate the Z-level and the rotational conformation achieved.

Amidst the 270 successful switch attempts, we failed to observe the Li-cage coordination of Z-level 6 (i.e. the Li atom closest to the metal surface) for the m-C_6 minority species. This can easily be rationalized by considering the probabilities involved: Only 9% of all Li@C_{60} molecules are adsorbed in the native m-C_6 orientation, and switching into Level 6 amounts to 2% of all excitation events measured. The discrete nature of the 14 Li-cage coordinations recorded for both majority and minority Li@C_{60} molecules rules out the possibility that the fullerene cage rotates (to any other coordination than C_6) during the excitation event.

Note that the 3 orientations of level 2 are mirror-symmetric for the M-C_6 and m-C_6 species. The same applies to level 5. In contrast, the 6 orientations of level 3/4 coincide for both M-C_6 and m-C_6 species. However, detailed analysis reported in **Supplementary Note 7** shows that the M-C_6 and m-C_6 species can be distinguished based on the characteristic kidney bean shape appearance of level 3/4 in STM images.

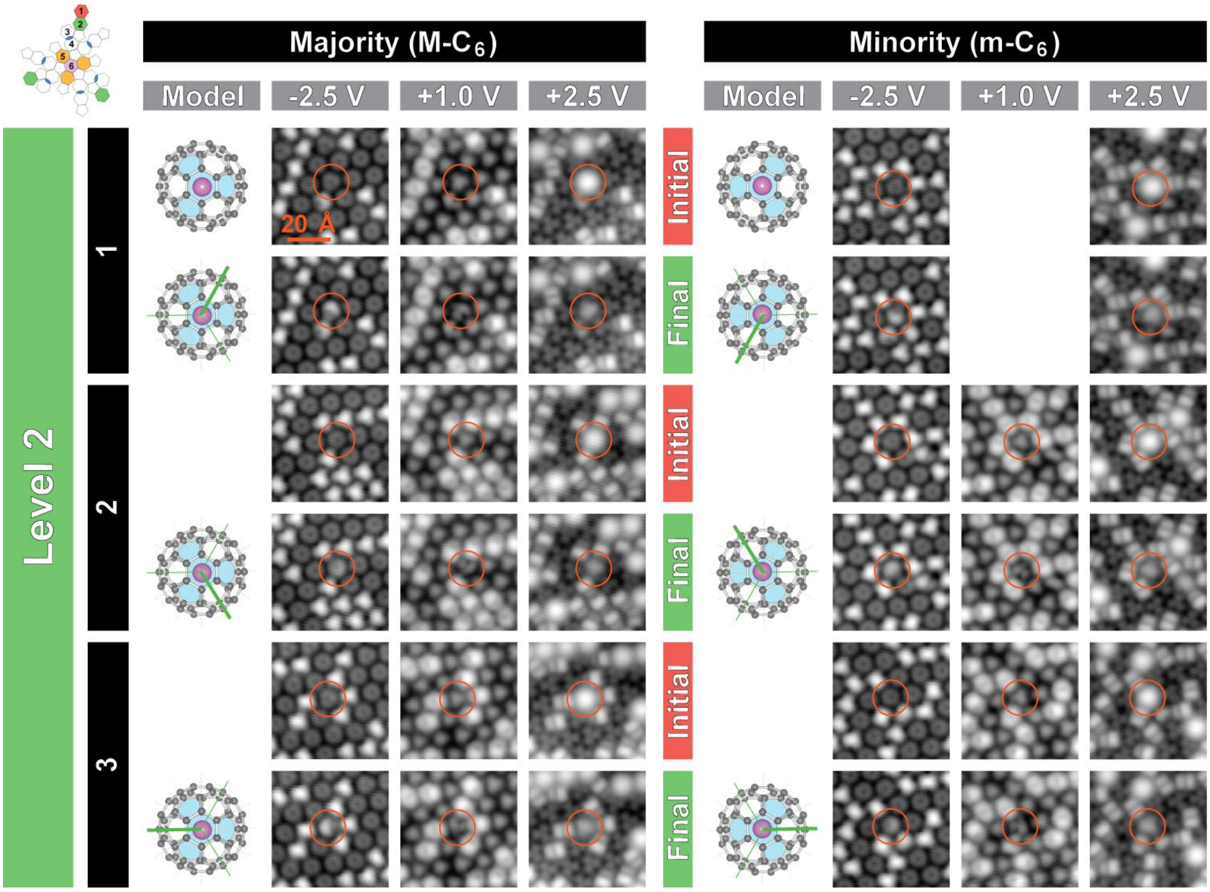

Supplementary Figure 6. Full collection of STM images identifying the 14 Li-cage coordinations for Li@C_{60}. Both M-C_6 and m-C_6 species are represented.

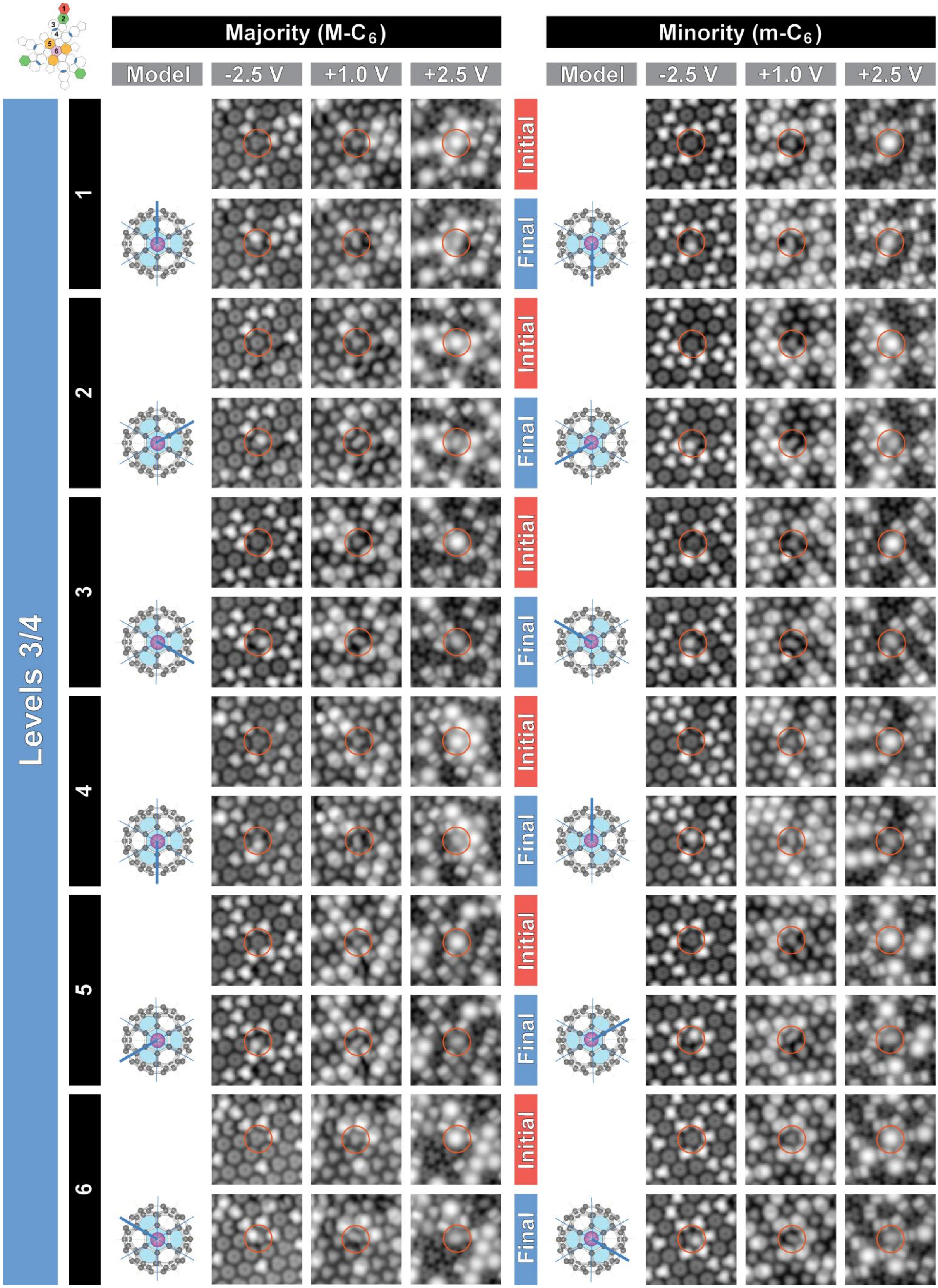

Supplementary Figure 6. Continued.

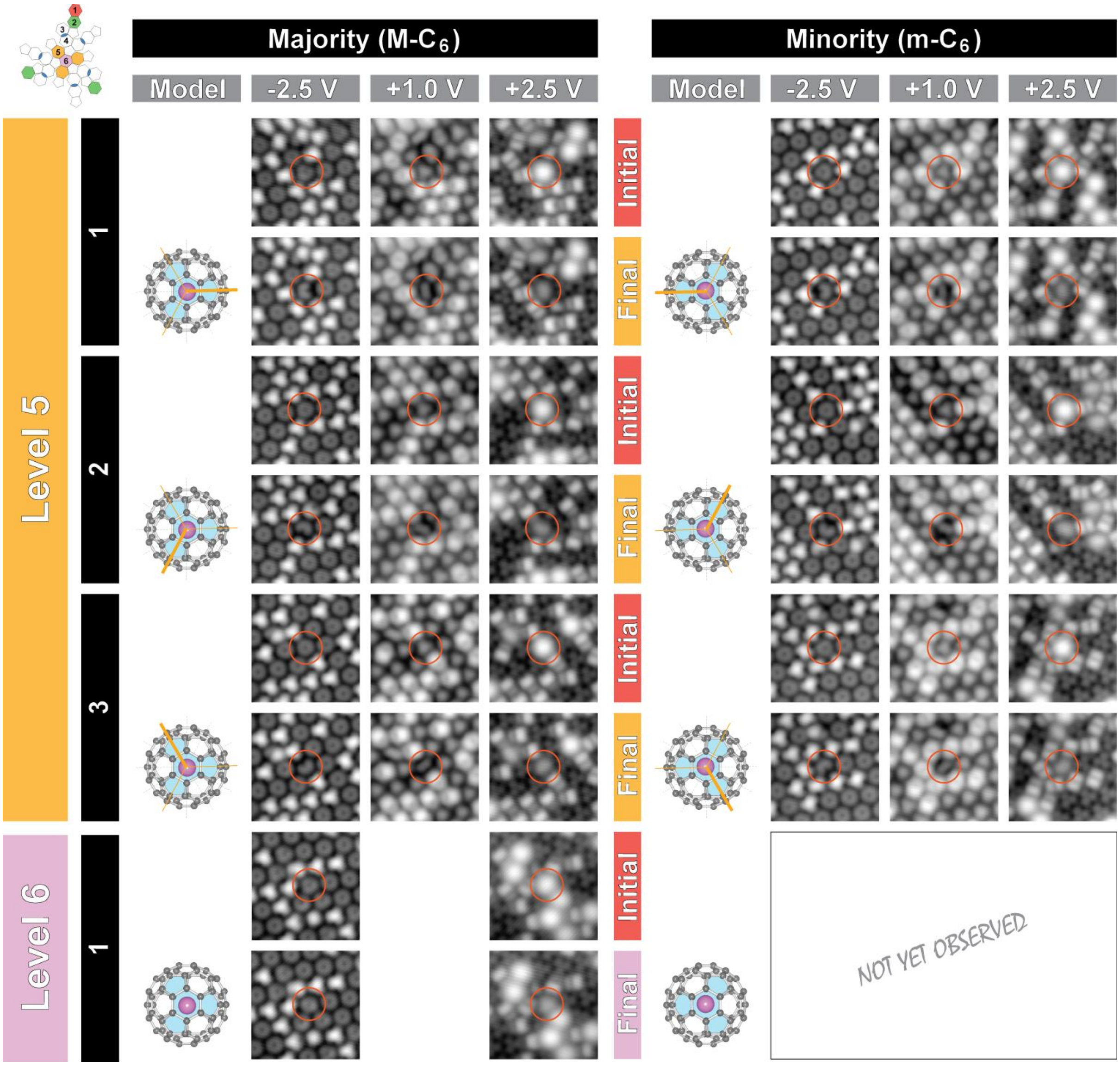

Supplementary Figure 6. Continued.

Supplementary Note 6. Induced neighbour rotations.

Consequence of neighbour cage rotations on *I(t)* spectra:

The excitation of Li@C_{60} molecules with pulses of +5 V and ca. 2 μA, which lead to Li-cage coordination switching, also occasionally induce the rotation of neighbouring empty C_{60} molecules. Examples of induced neighbour rotations are displayed in the sequential excitation procedure reported in **Supplementary Note 3**. **Figure S2(b)** shows that a C-apex molecule rotates to a C_6-C_6 configuration. **Supplementary Figure 3(f)** shows two nearest neighbours rotating into different configurations: One molecule rotates to a very slightly tilted M-C_6 coordination, and one molecule rotates to a (slightly off) C-apex configuration. **Supplementary Figure 3(i)** shows a C-apex molecule rotating 180° around the surface normal. The rotation of nearest-neighbours can be expected to lead to small (but measurable) changes in conductance of the target Li@C_{60} molecule due to alterations in the lateral intermolecular interactions. **Supplementary Figure 7** displays a series of selected single-point *I(t)* spectra acquired on top of the target Li@C_{60} molecule whilst monitoring the excitation events displayed in **Supplementary Note 3**. The light and dark grey traces monitor the changes in conductance accompanying the Li-coordination switches from levels 1-to-2 (**Supplementary Figures 3(a)-(b)**) and from levels 1-to-5 (**Supplementary Figures 3(i)-(j)**), respectively. The red trace illustrates the changes in conductance accompanying the induced-rotations of the two nearest-neighbour empty C_{60} molecules in **Supplementary Figures 3(e)-(f)**. One sees that the changes in conductance associated with induced neighbour rotations are smaller than those accompanying Li-cage coordination switches, and can be both positive and negative.

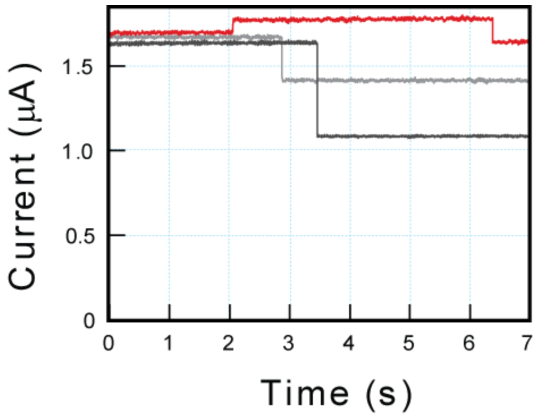

Supplementary Figure 7. Comparing the conductance changes due to ion-cage coordination switches and induced nearest-neighbour cage rotations. *I(t)* spectra reporting conduction changes of the target Li@C_{60} molecule associated with selected excitation events monitored in **Supplementary Note 3**. See text for details.

Lateral extent of neighbour cage rotations:

As discussed above, as well as in **Supplementary Note 3**, the excitation of a target Li@C_{60} molecule with 5.0 V and approximately 2 μA can also lead to the rotational activation of neighbouring C_{60} molecules. Shown in **Supplementary Figure 8** is an example of induced rotations at distances as large as 50 Å, equivalent to a separation accommodating 5 adjacent, close-packed molecules on the Au(111) surface. For this to happen, injected electrons must be capable of transporting over such large distances. The $P_{x,y}$-SAMOs afford an efficient electronic highway to do so if they undergo hybridisation with the quasi-degenerate $P_{x,y}$-SAMOs on the neighbouring C_{60}. Two-dimensional extended wave function delocalisation in pure C_{60} molecular islands via hybridisation of SAMOs was demonstrated by Feng *et al*. [**21**].

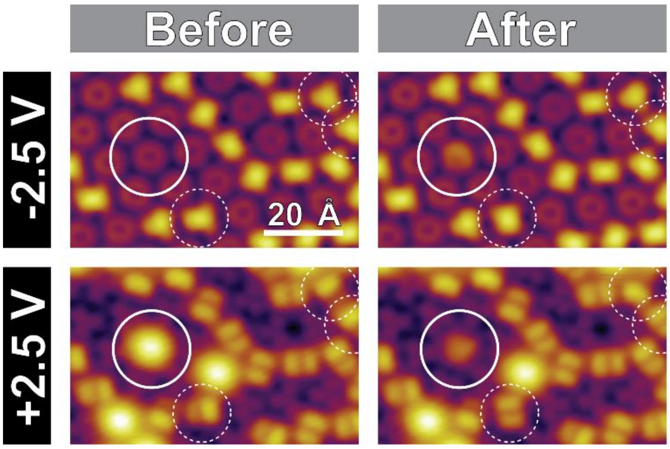

Supplementary Figure 8. Induced neighbour cage rotations. STM images acquired before and after excitation (5.0 V, 1.7 μA) of a Li@C_{60} molecule (thick white circle). The target molecule switches from level 1 to level 2. This is accompanied by rotations of three neighbouring C_{60} molecules, highlighted by the thin dashed circles.

Supplementary Note 7. Level 3/4 and its kidney bean shape.

As described in the main manuscript, we associate the kidney bean appearance of the switched Li@C_{60} to level 3/4. This level is proposed to arise from levels 3 and 4 which are not experimentally realised due to the subtle influence of intermolecular lateral interactions. For both M-C_6 and m-C_6 native Li@C_{60} species, 6 (not 6+6) distinct rotational orientations associated to level 3/4 are observed as presented in **Supplementary Note 5** and, with more detail, in **Supplementary Figure 9** below. Further support for the existence of level 3/4 is found by close inspection of the STM signatures of the kidney bean shape in relation to the various rotational alignments. The kidney bean shape is peculiar because of its lack of symmetry. The shape and its experimentally determined alignment are schematically illustrated in **Supplementary Figure 9** by the red (for the M-C_6 species) and blue (m-C_6) features for each rotational orientation. The shapes are exaggerated to show the orientation of high and low electron density regions in each feature. By looking at the STM images, and the associated shapes, one easily observes that the kidney bean alignment for a given rotational orientation is different for the M-C_6 and m-C_6 species, allowing to identify and discriminate between these species easily. As expected, a 180° rotation of M-C_6 reproduces the m-C_6 species. This indicates that the actual symmetry of level 3/4 is three-fold and not six-fold, as can be seen from the red and blue features. Three states are 120° rotationally equivalent and the other three are reflections of those, with the mirror axes matching the high symmetry directions of the molecular island. Upon close inspection of the C_{60} cage, the symmetry reduction is due to the alternating angle of the six C=C double bonds around its equator, the feature of the cage that the Li stabilises closest to in level 3/4. These bonds are highlighted by the blue ovals in the ball and stick models below. These models are illustrated such that the direction of viewing matches the axis of the kidney bean, with the highlighted bond coinciding with the bright area in the STM image. The coloured faces refer to the other Z-levels and are highlighted merely to aid in recognising the orientation of the molecule.

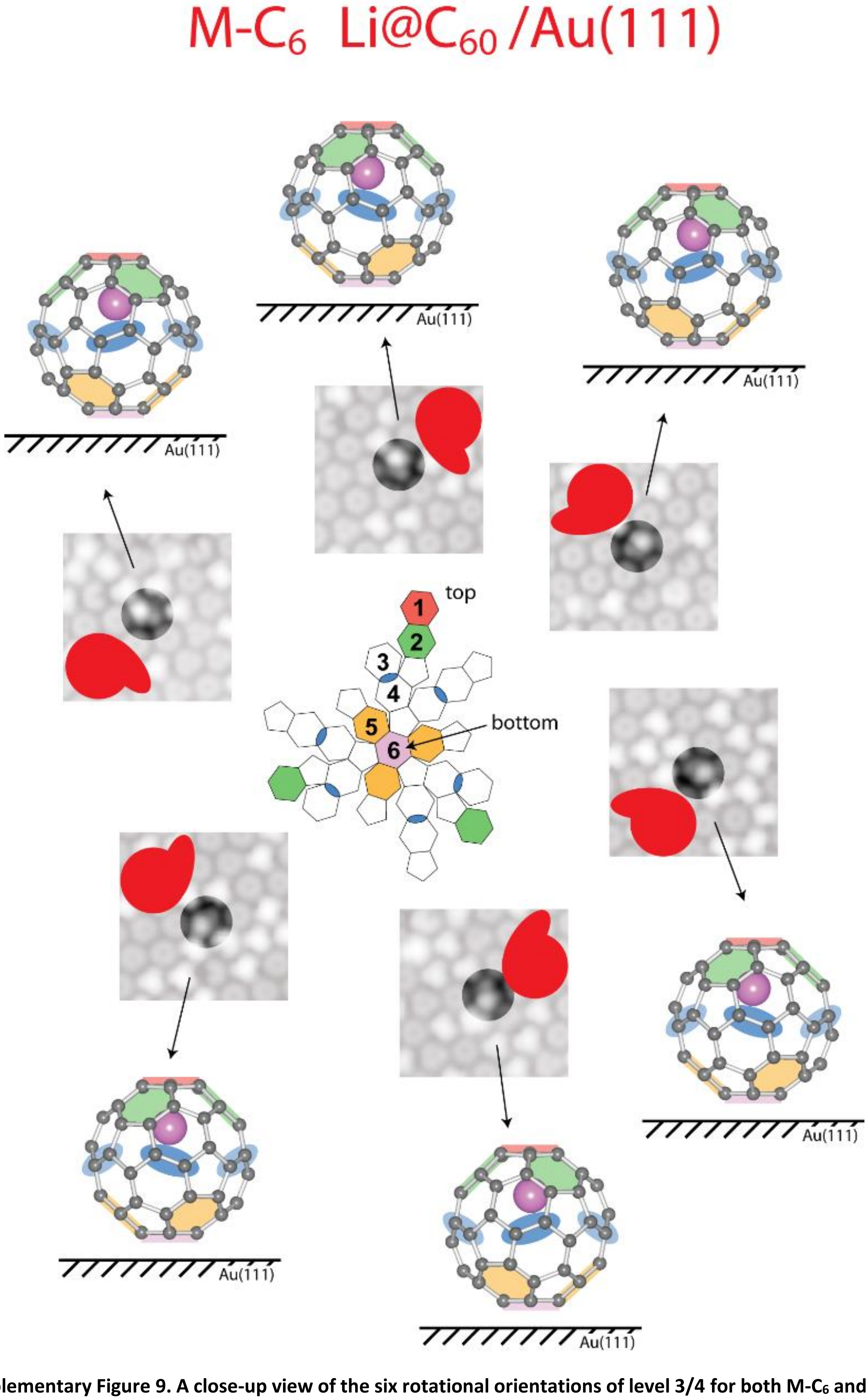

Supplementary Figure 9. A close-up view of the six rotational orientations of level 3/4 for both M-C_6 and m-C_6 native Li@C_{60} species. The STM images are acquired at –2.5V and 0.1 nA.

m-C_6 Li@C_{60} /Au(111)

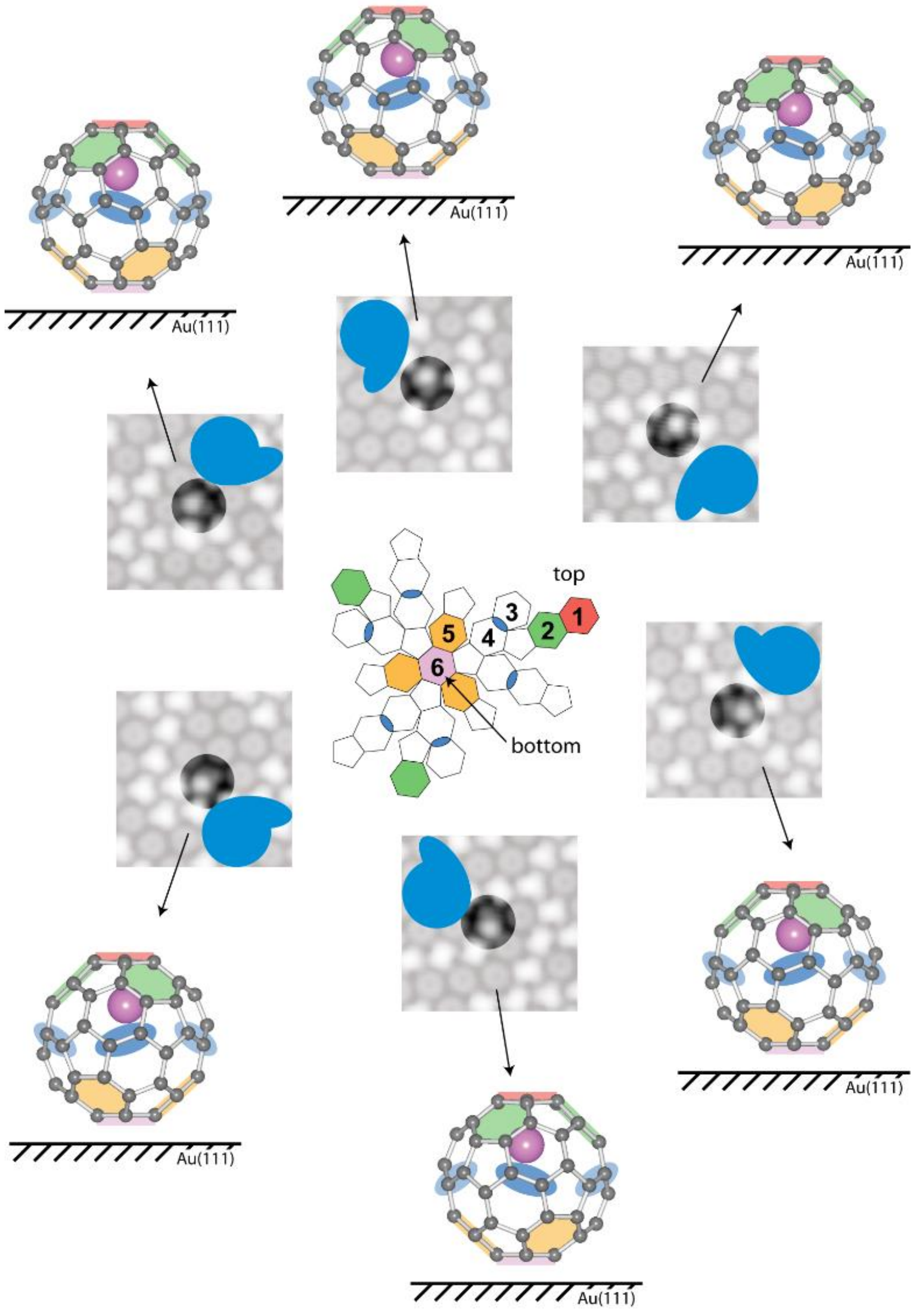

Supplementary Figure 9. Continued.

Supplementary Note 8. Conductance versus time spectra acquired during switching.

We start here by considering the *I(t)* spectrum displayed in **Figure 3(a)** exhibiting several distinct conductance levels. Amongst these, some are seen to show relatively similar (but discernible) conductance, whereas others are very different. The association of a given conductance level to a given Li-cage coordination is not straightforward. Indeed, the excitation of a Li@C_{60} molecule with a 5 V pulse of 2 μA leads very often to rotations of neighbouring empty C_{60} molecules. As seen in the data reported in **Supplementary Notes 3** and **6**, neighbouring molecules are seen to rotate under the influence of the STM tip excitation (we have no evidence that the target Li@C_{60} cage rotates, and we never witnessed a Li@C_{60} being switched at distance). The rotational excitations of C_{60} closest-neighbours induce minor electronic alterations in the target Li@C_{60} due to subtle changes in lateral intermolecular interactions, and manifest as small conductance changes similar to those observed in **Figure 3(a)** (see **Supplementary Note 6**). We ascribe, however, the larger conductance changes to Li-cage coordination switches. In order to investigate the conductance changes involved upon switching from one Z-level to the other, we have acquired *I(t)* spectra for each of the 270 switching examples discussed above (similar to the inset of **Figure 3(h)**). Selected spectra (those that are unperturbed by alterations to direct neighbours) are reported in **Supplementary Figure 10**. Since all initial configurations refer to the same Z-level (level 1 with native M-C_6), we have normalised all *I(t)* traces (red portion of the traces prior to switch event, $t < 0$). Post-switch ($t > 0$), the *I(t)* traces are coloured with reference to the resulting final ion-cage state (inferred from the STM images described in **Figures 4(a)-(e)** and **Supplementary Note 5**). One can see that some Z-levels (1, 5, and 6), but not all, can be differentiated based on their conductance. Levels 2 and 3/4 generate a fairly broad band of partially intermingled conduction traces, hence these levels cannot be clearly discriminated based on their conductance alone, but can be discriminated based on the energy of their P_z-SAMO resonance in constant-current *dI/dV* spectra (see blue and green traces in **Figure 4(f)**) and their topographic appearance in STM images (**Figures 4(b)** and **(c)**). Thanks to **Supplementary Figure 10**, a detailed description of **Figure 3(a)** can be made. The smaller conductance changes can be associated to induced nearest-neighbour rotations, whilst the larger *I(t)* changes relate to Li-cage coordination switches. Hence, **Figure 3(a)** can be said to capture four conductance levels relating to different Li-cage coordinations. Furthermore, a tentative assignment to Z-levels can be made: From high to low conductance, the Z-levels monitored are 1, 2/3/4 (these three levels remain indistinguishable in their conductance), 6, and 5, respectively.

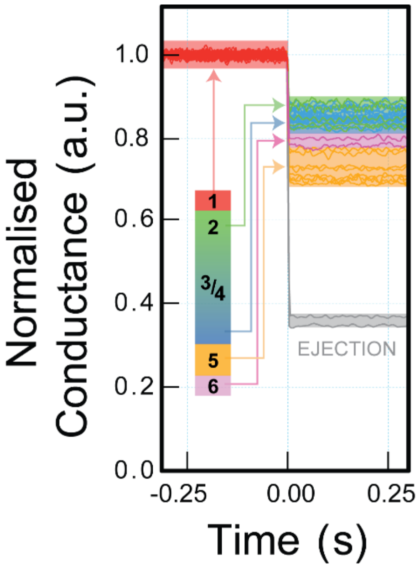

Supplementary Figure 10. Selected *I(t)* spectra acquired during the 270 successful switching measurements. All spectra are normalised to allow for comparison. In addition, two spectra following Li ejection events are reported (in grey).

We finally note that different STM tips lead to different magnitudes of the conductance changes recorded. However, the consistency across data sets is always guaranteed on a relative basis. That is, we have always observed that the sequence from 1 to 2/3/4 to 6 to 5 is preserved with decreasing conductivities as shown in **Supplementary Figure 10**. Hence, the data set displayed in **Supplementary Figure 10** was acquired over a given period of time with only minor STM tips changes.

Supplementary References.

[1] M. Stefanou, H. J. Chandler, B. Mignolet, E. Williams, S. A. Nanoh, J. O. F. Thompson, F. Remacle, R. Schaub, E. E. B. Campbell. Angle-resolved photoelectron spectroscopy and scanning tunnelling spectroscopy studies of the endohedral fullerene Li@C_{60}. *Nanoscale* **11**, 2668–2678 (2019).

[2] J. A. Gardener, G. A. D. Briggs, M. R. Castell. Scanning tunneling microscopy studies of C_{60} monolayers on Au(111). *Physical Review B* **80**, 235434 (2009).

[3] M. De Menech, U. Saalmann, M. E. Garcia. Energy-resolved STM mapping of C_{60} on metal surfaces: A theoretical study. *Physical Review B* **73**, 155407 (2006).

[4] X. Lu, M. Grobis, K. H. Khoo, S. G. Louie, M. F. Crommie. Charge transfer and screening in individual C_{60} molecules on metal substrates: A scanning tunneling spectroscopy and theoretical study. *Physical Review B* **70**, 115418 (2004).

[5] G. Schull, N. Néel, M. Becker, J. Kröger, R. Berndt. Spatially resolved conductance of oriented C_{60}. *New Journal of Physics* **10**, 065012 (2008).

[6] W. Auwärter, K. Seufert, F. Bischoff, D. Ecija, S. Vijayaraghavan, S. Joshi, F. Klappenberger, N. Samudrala, J. V. Barth. A surface-anchored molecular four-level conductance switch based on single proton transfer. *Nature Nanotechnology* **7**, 41–46 (2012).

[7] T. Huang, J. Zhao, M. Feng, A. A. Popov, S. Yang, L. Dunsch, H. Petek. A Molecular Switch Based on Current-Driven Rotation of an Encapsulated Cluster within a Fullerene Cage. *Nano Letters* **11**, 5327–5332 (2011).

[8] D. M. Eigler, C. P. Lutz, W. E. Rudge. An atomic switch realized with the scanning tunnelling microscope. *Nature* **352**, 600–603 (1991).

[9] P. Liljeroth, J. Repp, G. Meyer. Current-Induced Hydrogen Tautomerization and Conductance Switching of Naphthalocyanine Molecules. *Science* **317**, 1203–1206 (2007).

[10] Y. Yasutake, Z. Shi, T. Okazaki, H. Shinohara, Y. Majima. Single Molecular Orientation Switching of an Endohedral Metallofullerene. *Nano Letters* **5**, 1057–1060 (2005).

[11] G. J. Simpson, S. W. L. Hogan, M. Caffio, C. J. Adams, H. Früchtl, T. van Mourik, R. Schaub. New Class of Metal Bound Molecular Switches Involving H-Tautomerism. *Nano Letters* **14**, 634–639 (2014).

[12] K. Morgenstern. Switching individual molecules by light and electrons: From isomerisation to chirality flip. *Progress in Surface Science* **86**, 115–161 (2011).

[13] G. Schulze, K. J. Franke, A. Gagliardi, G. Romano, C. S. Lin, A. L. Rosa, T. A. Niehaus, T. Frauenheim, A. Di Carlo, A. Pecchia, J. I. Pascual. Resonant Electron Heating and Molecular Phonon Cooling in Single C_{60} Junctions. *Physical Review Letters* **100**, 136801 (2008).

[14] G. Schulze, K. J. Franke, J. I. Pascual. Resonant heating and substrate-mediated cooling of a single C_{60} molecule in a tunnel junction. *New Journal of Physics* **10**, 065005 (2008).

[15] Z. Slanina, F. Uhlik, S.-L. Lee, L. Adamowicz, S. Nagase. MPWB1K calculations of stepwise encapsulations: Li_x@C_{60}. *Chemical Physics Letters* **463**, 121–123 (2008).

[16] B. I. Dunlap, J. L. Ballester, P. P. Schmidt. Interactions between C_{60} and Endohedral Alkali Atoms. *Journal of Physical Chemistry* **96**, 9781–9787 (1992).

[17] E. E. B. Campbell, M. Fanti, I. V. Hertel, R. Mitzner, F. Zerbetto. The hyperpolarisability of an endohedral fullerene: Li@C_{60}. *Chemical Physics Letters* **288**, 131–137 (1998).

[18] Y. S. Li, D. Tománek. How free are encapsulated atoms in C_{60}. *Chemical Physics Letters* **221**, 453–458 (1994).

[19] S. Aoyagi, E. Nishibori, H. Sawa, K. Sugimoto, M. Takata, Y. Miyata, R. Kitaura, H. Shinohara, H. Okada, T. Sakai, Y. Ono, K. Kawachi, K. Yokoo, S. Ono, K. Omote, Y. Kasama, S. Ishikawa, T. Komuro, H. Tobita. A layered ionic crystal of polar Li@C_{60} superatoms. *Nature Chemistry* **2**, 678–683 (2010).

[20] X. Torrelles, M. Pedio, C. Cepek, R. Felici. (2√3×2√3)R30 induced self-assembly ordering by C_{60} on a Au(111) surface: X-ray diffraction structure analysis. *Physical Review B* **86**, 075461 (2012).

[21] M. Feng, J. Zhao, H. Petek. Atomlike, Hollow-Core–Bound Molecular Orbitals of C_{60}. *Science* **320**, 359–362 (2008).
