# Supplementary material for: Li@C60 as a multi-state molecular switch
Source: Nat Commun. 2019 May 23;10:2283. doi: 10.1038/s41467-019-10300-2 (PMC6533348; doi:10.1038/s41467-019-10300-2)
Supplement: Supplementary file 2 — Peer Review [file 41467_2019_10300_MOESM2_ESM.pdf]

Reviewers' comments:

Reviewer #1 (Remarks to the Author):

The manuscripts describes in-depth low-temperature STM study of Li@C60. The authors demonstrate that position of Li ion inside the fullerene cage can be switched by tunneling current at high positive bias voltage, and localize several well defined position of Li, which can be achieved as a result of such switching events. Each position is characterized by characteristic topography as well SAMO energy distribution, which is noticeably different from the ground state. This is a significant achievement in creating of single-molecule switches (although one has to acknowledge than the switching happens at rather extreme conditions). The work is carefully performed and results are analyzed exhaustively. I believe the work will be of high interest for readers of Nat. Commun. and recommend publication after the authors clarify the points outlined below.

1. Taking into account that initial sample contained 80% of Li@C60, the low content of Li@C60 in the monolayer (ca 13%) is rather surprising and requires additional clarification

2. What is the state of Li@C60 on the surface? Is it neutral molecule? Or counter-ions are still somewhere around? Neutral Li@C60 is known to be very chemically active. For instance, it dimerizes spontaneously as shown in Chem. Sci. 2016, 7, 5770. It may also interact with neighboring C60 molecules. Could the authors also observe the fragment with two neighboring Li@C60 molecules? If C60 is more volatile as it seems, it may be possible to anneal the substrate with C60/Li@C60 islands to induce partial evaporation of C60, which should increase the surface concentration of Li@C60.

3. Intermolecular hybridization of SAMOs is not considered in sufficient details (except for mentioning of possible band formation). Two STM studies of SAMOs in metallofullerenes are relevant for the discussion of this paper.
Phys. Rev. B. 2010 81, 085434 (SAMOs of Sc3N@C80 and intermolecular hybridization)
Phys. Rev. B. 2013 88, 075417 (SAMO in La@C82 – energy stabilization)
In particular, the question how intermolecular hybridization affects the situation and what is changing for intermolecular hybridization when Li is switching between different positions is not discussed. For instance, did the authors analyzed dI/dV spectra of neighboring C60 molecules after switching event in Li@C60.

4. It would be important to try to perform the measurement for single Li@C60 molecules

5. A small technical moment – Schlegel diagram is a convenient and well accepted way of plotting C60 in 2D. The use of Schlegel diagrams in Fig. 1b and Fig. 4g would make it easier for a reader

6. Figure 4 there is a mistake with "f" and "g", I guess they are swapped

Reviewer #2 (Remarks to the Author):

Chandler et al present an STM investigation of tunneling electron current switching of single Li@C60 molecules. The results are interesting and should be published. Having looked at the background literature, I have a few quibbles that should be addressed before the publication.

It appears that a related isomerization mechanism was presented by Jorn et al. (Ref. 24) using light to populate the SAMO electronic state rather than tunneling electrons. It seems appropriate that this theory should be given more prominence, since it was predicted a long time ago that populating the SAMO state might induce switching. What is interesting is that the s-SAMO acceptor state is not very efficient for switching, but p-SAMOs at higher energies are better. This is interesting because in Ref.

25 it is predicted that s-SAMO is more stabilized than p-SAMO with the Li atom in the center, hence there should be a stronger driving force for the isomerization. Although the authors suggest that s-SAMO may be strongly interacting with the substrate, according to the supplementary materials it is obvious that this should be strongly dependent on the isomer. Perhaps a more likely situation is that s-SAMO makes a highly delocalized band, so the energy may be delocalized among many molecules.

Furthermore, the authors make a big point that their molecule has 14 identifiable isomers, which potentially makes it superior to other molecules as a molecular switch. What they neglect to emphasize is that in their system switching occurs randomly between the 14 isomers at a rather high voltage. By contrast in Ref. 2, it is claimed that switching in a similar system occurs deterministically between 3 different pairs of states, which also seems to have substantial merit. For some material involving isomerization of endohedral fullerenes to achieve a realistic application, it is probably more advantageous to present a more comprehensive discussion, rather than to claim some questionable superiority of one material over another.

With same goal, it is probably useful to cite work where superatom states are introduced to a broad audience, such as Acc. Chem. Res. 44, 360 (2011), since the concept is general but may not be very familiar.

Reviewer #3 (Remarks to the Author):

First, my apologies for the delay in returning this review.

I enjoyed this paper very much. It's a tour de force analysis of very high quality STM images and dI/dV spectra of Li@C60 on Au(111). As with most single molecule STM papers claiming a new advance in molecular electronics, this is exciting fundamental research that is unlikely to revolutionise the ICT sector any time soon. The lack of direct application, however, is not a criticism.

For me, the more exciting aspect of this work is the ability to explore the potential energy landscape experienced by a species within a fullerene cage in a highly reproducible and rather elegant fashion. On first reading the paper, I was unconvinced that the authors had indeed targetted the SAMO orbitals as they suggest but I became much more convinced as I failed to find holes in their arguments on a second reading.

I recommend this paper for publication following only the minor changes listed below:

1. "The following work demonstrates for the first time 14-state switching behaviour of individual Li@C60 molecules condensed on a Au(111) surface using low temperature, ultrahigh-vacuum scanning tunnelling microscopy and spectroscopy. "

This is not at all the most compelling closing line for the introduction. It's not so surprising that this has been achieved "for the first time" given the narrowness of the system as described. The much more general result is that the the authors have succeeded in exploring the potential energy landscape inside an endofullerene via careful and sophisticated analysis of variations in SAMO orbitals.

2. In Fig.2 (and other figures in the paper), the STM bias voltage is +2.5 V and yet the key SAMO resonance is at 2.1 V. The obvious question to ask is why "spectroscopic imaging" wasn't carried out at 2.1 V so as maximise the contribution of that SAMO state to the images? A constant height dI/dV map acquired at 2.1 V would be very interesting to see. Do the authors have these data, or could they quickly acquire an image of this type to add to the paper?

3. What's intriguing is that the Li never seems to reach a global minimum? Am I correct in saying this or do I misunderstand the authors' conclusions? Do the authors have any evidence (from, e.g. a combination of DFT and NEB barrier calculations) for the depth of the various potential minimia in the cage?

4. The labelling of Fig.4 (f) and Fig. 4(g) is the wrong way round.

5. I think it's a "reach" to claim that Fig. 5 is direct evidence of Li ejection. There is no direct chemical information in the images. A highly localised (!) photoemission experiment could provide that direct information but the claim that Li has been ejected relies on the authors' interpretation of the data. It's certainly strong, credible evidence but I don't think it can be described as "direct".

6. Inelastic tunnelling spectroscopy could in principle be helpful in monitoring the vibrational modes of the Li. Have the authors attempted this measurement?

I am waiving my right to anonymity for this review.

Philip Moriarty, School of Physics and Astronomy, University of Nottingham

We thank our three reviewers for their diligent assessment of our manuscript, and for recognising our results and analysis are of very high quality. We are grateful for the opportunity to make a few minor corrections so that it may be published in Nature Communications, following the three reviewers' recommendation. We are also grateful to Dr Ariane Vartanian, the Senior Editor, for the opportunity to respond to the comments made by the reviewers, which can be found below.

(All references below refer to the updated reference numbering.)

REVIEWER #1:

Reviewer #1 recommends our paper for publication after clarification of the points outlined below.

- **Taking into account that initial sample contained 80% of Li@C_{60}, the low content of Li@C_{60} in the monolayer (ca 13%) is rather surprising and requires additional clarification.**
  - → On page 4 of the manuscript, last line, we explain why we observe a loss of Li: "*The presence of empty C_{60} on the substrate is attributed to decay processes occurring in the molecular evaporator and is consistent with the presence of C_{60} in the gas phase mass spectra obtained under similar annealing and evaporation treatment [35]*." Reference [35] relates to our previous work which reports thorough details on our explanation about the Li loss as quantified from photoelectron spectroscopy and mass spectroscopy measurements, the details were given in the Supplementary Information provided with that publication [35]. The loss of Li when evaporating the material is also consistent with results reported previously by Yamada *et al*. who have shown Li loss on evaporation of Li@C_{60} under similar conditions, listed as reference [26].
  - → Prior to any deposition, we anneal the molecules in our oven (a home-built evaporator) in order to degas and remove impurities and ligands. This conditioning process is accompanied with a slow thermal decay corresponding to loss of Li content. The annealing condition were chosen to be similar to those used prior to the gas phase experiments (reference [35]) to ensure the presence of clean fullerene deposits (and to provide a check on the Li@C_{60}/C_{60} ratio that was present in the gas phase target). It is possible to increase the content of Li@C_{60} by annealing for a shorter time, using increased flash annealing at the substrate for additional cleaning, but that was not felt to be necessary for the reported experiments where we are interested in the switching behaviour of individual molecules on the substrate.
  - → In order to clarify this further, we have added the following sentence in the methodology section (page 3): "In this conditioning phase prior to deposition, some Li content is lost through a slow thermal decay process [**26, 35**]."

- **What is the state of Li@C_{60} on the surface? Is it neutral molecule? Or counter-ions are still somewhere around? Neutral Li@C_{60} is known to be very chemically active.**

For instance, it dimerizes spontaneously as shown in Chem. Sci. 2016, 7, 5770. It may also interact with neighboring C_{60} molecules. Could the authors also observe the fragment with two neighboring Li@C_{60} molecules?

→ We have no evidence from our mass spectroscopy experiments, extensively reported in reference [35], that positive Li@C_{60} are emitted from our oven. It follows unambiguously from that work that we must have neutral species since we are photo ionising the molecules that sublime from the oven to produce singly charged ions (see [35]). There is also evidence from Yagi *et al*. [Chemical Physics Letters **651**, 124-126 (2016)] that the Li@C_{60} material that is deposited in their experiment is neutral with a partial charge transfer from the Li to the carbon cage giving a signal in the XPS similar to that seen for C_{60} anions and they also determined the shift in the UPS spectral onset compared to C_{60} that was consistent with a shift in the ionisation energy of ca 1.1 eV downwards compared to neutral C_{60} as expected for the neutral molecule (a result of the at least partial charge transfer between Li and the cage).

→ Hence, when deposited on our metallic substrate, Au(111), we are confident the Li@C_{60} is neutral.

→ Both Yagi *et al.* [see above] and Yamada *et al.* (reference [26]) discuss the removal of PF_6- on heating/evaporating. In addition, after molecular deposition, we anneal our substrate to selectively desorb any ligand/pollution and create large, well-ordered islands of fullerene cages. We have no evidence of ligands remaining on the surface after our preparation procedure.

→ The tendency of neutral Li@C_{60} to oligomerise is also well-known from the early experiments carried out by one of the current authors (EEBC) that showed the presence of dimers and trimers when the material was extracted from Li^+-irradiated C_{60} films by dissolving in organic solvent, without forming a stabilising ionic complex with PF_6^- and purifying by HPLC [Gromov *et al*., J. Phys. Chem. B **107**, 11290 (2003)]. The conditions in the current experiment are very different to both of these studies (room temperature and in the presence of solvents and other species). In our case, the monomeric material is evaporated and deposited on a cold substrate held at liquid He temperature, with a relatively low content (13%) of Li@C_{60}. We have not found any evidence for dimer formation so far in our STM studies.

- **If C_{60} is more volatile as it seems, it may be possible to anneal the substrate with C_{60}/Li@C_{60} islands to induce partial evaporation of C_{60}, which should increase the surface concentration of Li@C_{60}.**

  → Actually from comparative studies that have been carried out (partially shown in Fig. S3 in the SI attached to reference [35]), the volatility of fullerenes from a pure C_{60} sample and from a Li@C_{60} sample appears to be very similar based on studies where the ion yield was monitored as a function of oven temperature in a mass spectrometry experiment. The decreased ratio of Li@C_{60} to C_{60} in the deposited material is probably not due to the increased volatility of any C_{60} impurities in the original material (or formed on decomposition) but to the removal of Li from the fullerene cage in the hot oven environment in the presence of solvent and ligand material.

- → The referee is, however, correct in suggesting it may be possible to selectively desorb the C_{60} from the surface since C_{60} is less strongly bound to the Au than the Li@C_{60}. We did not explore this option as it was outwith the scope of this paper. We however point out that we do not know if annealing at elevated temperatures would lead first to C_{60} sublimation from the surface (thereby increasing the Li@C_{60} surface concentration), or first to Li decay from the Li@C_{60} cages (thereby increasing the C_{60} surface concentration even further). We note that, in line with the referee's suggestion, we have nevertheless demonstrated in reference [35] that, by annealing at moderate temperatures, we can partially segregate Li@C_{60} and C_{60} due to their different diffusivities.

- **Intermolecular hybridization of SAMOs is not considered in sufficient details (except for mentioning of possible band formation). Two STM studies of SAMOs in metallofullerenes are relevant for the discussion of this paper. Phys. Rev. B. 2010 81, 085434 (SAMOs of $Sc_3N@C_{80}$ and intermolecular hybridization) Phys. Rev. B. 2013 88, 075417 (SAMO in La@C_{82} – energy stabilization). In particular, the question how intermolecular hybridization affects the situation and what is changing for intermolecular hybridization when Li is switching between different positions is not discussed. For instance, did the authors analyzed dI/dV spectra of neighboring C_{60} molecules after switching event in Li@C_{60}.**
  - → Intermolecular hybridisation has been considered in our work in that this is responsible for limiting the 20 possible ion-cage coordinations to only the 14 measurable. This is discussed e.g. in page 11 of our manuscript.
  - → Investigating how intermolecular hybridisation changes as a function of Li position is simply well beyond the scope of this paper. We note here that we have 14 different isomers, rendering any viable statistical analysis extremely time consuming.
  - → We have not explicitly analysed dI/dV spectra of neighbouring C_{60} molecules after switching events in Li@C_{60}. However, along the lines the referee is suggesting here, we have reported in Fig. S3 dI/dV spectra acquired over many Li@C_{60} molecules. For instance, the red traces relate to Li@C_{60} in their native state. These spectra are extremely reproducible. Hence these spectra do not exhibit any visible differences (in their LUMO and SAMO orbitals) that could be ascribed to varying intermolecular hybridisation, and yet, the molecules have different nearest neighbours (from 0 to 6 surrounding Li@C_{60} molecules, the rest being C_{60}).

- **It would be important to try to perform the measurement for single Li@C60 molecules.**
  - → We have attempted such measurements. Performing switching measurements on isolated molecules (we show in reference [35] how to prepare isolated Li@C_{60}) is much more demanding and likely impossible as explained here:
    - Due to the 3d nature of the isolated molecule (as opposed to the 2d overlayer when in islands), it is very difficult to assess if a molecule switches or not based on topography alone. The topographic signature of an isolated Li@C_{60} is just a big bump – see e.g. Fig. 10 of ref [35] – from which subtle changes due to isomerisation can hardly be identified.

- More often than not, a molecule will undergo STM-tip induced diffusion upon switching excitation. This is clearly hindered when the cages are located within molecular islands, as we demonstrate in our paper.

→ These difficulties have prevented us from making a systematic analysis of switching in isolated molecules. We have hence not reported on our attempts. However, we can anticipate that, if switching in isolated molecules is feasible, then 20 states (not the 14 described in our manuscript for molecules within islands, due to nearest neighbour influences) could possibly be observed.

→ To clarify this in the manuscript, we have added the following sentence (last line of the conclusion, page 14): Further exploration of the isomerisation mechanism could be applied to isolated Li@C_{60} molecules. We suspect, however, that if the manipulation parameters didn't merely result in molecular diffusion, we could expect 20 distinct cage coordinations due to the lack of neighbouring fullerenes affecting the internal surface potential of the Li@C_{60}.

- **A small technical moment – Schlegel diagram is a convenient and well accepted way of plotting C_{60} in 2D. The use of Schlegel diagrams in Fig. 1b and Fig. 4g would make it easier for a reader.**

  → We thank the referee for the suggestion. We initially considered the Schlegel diagram but decided the distortions to the hexagonal faces were unhelpful. However, we have now included Schlegel diagrams in the supplementary material (see section SI.8. with Fig. S10) to act as alternative diagrams for those who are more familiar with the Schlegel representation. We have added the following sentence in the caption of Fig. 1: "For a colour-coded Schlegel diagram of a C_{60} see **SI.8**."

- **Figure 4 there is a mistake with "f" and "g", I guess they are swapped.**

  → We thank the referee for noting this error. We have fixed it in the manuscript.

REVIEWER #2:

Reviewer #2 comments "*The results are interesting and should be published*".

- **It appears that a related isomerization mechanism was presented by Jorn et al. (Ref. 24) using light to populate the SAMO electronic state rather than tunneling electrons. It seems appropriate that this theory should be given more prominence, since it was predicted a long time ago that populating the SAMO state might induce switching.**

  → Reference 24 (now [22] in the revised version) is a theoretical investigation of current-driven dynamics. There is just a speculative comment at the end that fs lasers coupled to STM may be able to measure the effects that they predict. We are of course aware and acknowledge that Petek and co-workers, who have forged

much of our current understanding of SAMOs as studied by STM, have predicted (e.g. in reference [22]) the potential role of the S-SAMO orbital as a means for Li activation within the fullerene cage (isomerisation). In order to emphasise their pioneering contributions we have added the red text to the conclusion of our manuscript: "We propose that Li activation is a result of resonant tunnelling via SAMO resonances, as initially suggested in a theoretical study by Jorn *et al*. [**22**]."

- **What is interesting is that the s-SAMO acceptor state is not very efficient for switching, but p-SAMOs at higher energies are better. This is interesting because in Ref. 25 it is predicted that s-SAMO is more stabilized than p-SAMO with the Li atom in the center, hence there should be a stronger driving force for the isomerization.**
  - → We agree with the referee that these observations are interesting and form the basis of our proposed Li activation mechanism. We however note that our recent combined photoemission + STM + tdDFT work [35] indicates that the P_z-SAMO becomes more stabilised than the S-SAMO due to the off-centre ground-state position of Li within the carbon cage. Yet, no isomerisation is observed for energy conditions involving the P_z - nor the S-SAMOs. Activation energies correlate with the occurrence of the $P_{x,y}$-SAMOs located above the P_z- and S-SAMOs.

- **Although the authors suggest that s-SAMO may be strongly interacting with the substrate, according to the supplementary materials it is obvious that this should be strongly dependent on the isomer. Perhaps a more likely situation is that s-SAMO makes a highly delocalized band, so the energy may be delocalized among many molecules.**
  - → The referee is correct to point out, as we do in our supplementary material, that the S-SAMO (as well as the P_z-SAMO) interaction with the substrate is strongly dependent on the isomer. The interaction of the S-SAMO and all other SAMOs has been assessed experimentally in Fig. 4f of our manuscript, by monitoring energy variations in the SAMO resonances as function of Li location (i.e. isomer). The experimental trends match what the theoretical predictions (Fig. S4) indicate. This correlation is further highlighted by Referee #3 who points out (see our reply below) that our work allows the potential energy landscape of the inner walls of the carbon cage to be examined by our spectroscopy measurements.
  - → We refute the argument put forward by the referee as a more likely situation. The energies of the S-SAMOs of Li@C_{60} (2.1 eV) and C_{60} (3.9 eV) do not overlap (see Fig. 2h). Hence the highly delocalised S-SAMO band arising from close-packed C_{60} is not well aligned with the S-SAMO resonance of the interspersed Li@C_{60} molecules. As a consequence of this, energy delocalisation is severely reduced. But most importantly, we highlight reference [46] in which the authors report on the theoretical prediction that hot electron cooling from the S-SAMO in a supported C_{60} system is significantly less efficient than interfacial charge transfer.

- **Furthermore, the authors make a big point that their molecule has 14 identifiable isomers, which potentially makes it superior to other molecules as a molecular switch. What they neglect to emphasize is that in their system switching occurs randomly between the 14 isomers at a rather high voltage. By contrast in Ref. 2, it is**

claimed that switching in a similar system occurs deterministically between 3 different pairs of states, which also seems to have substantial merit. For some material involving isomerization of endohedral fullerenes to achieve a realistic application, it is probably more advantageous to present a more comprehensive discussion, rather than to claim some questionable superiority of one material over another.

→ We agree with the referee that the switch we report here does not afford control over the final state (isomer), we have a statistical population of Li positions within the cage after switching as discussed on p. 9 of the manuscript. In ref 2 (now [7] in the revised version) where a Sc_3N molecule was rotated within C_{80} to produce identifiable isomers, the excitation mechanism was predominantly via the excitation of vibrational modes from inelastically scattered tunnel electrons, and thus at lower tip bias voltages. This provided a certain degree of control by providing dominant *stochastic* switching between isomers with low energy barriers when using low bias voltage. The authors claim *hierarchical* switching (stated in their summary on p. 5331 of that paper) based on the range of energetic barriers between possible rotational isomers and the dominance of low barrier switching for low bias voltages, not *deterministic* switching. So they do have some element of control but perhaps not as much as indicated by the referee.

→ Furthermore, we do not claim any superiority of materials. We thank the referee, however, for raising this issue. We have therefore brought several changes to the manuscript as follow:

→ In the abstract we changed "we report on a multi-state single molecule switch using the endohedral fullerene $Li@C_{60}$ that displays 14 accessible molecular states." to "we report on a multi-state single molecule switch using the endohedral fullerene $Li@C_{60}$ that displays 14 molecular states which can be statistically accessed."

→ In the conclusion we changed "By applying electric fields in excess of 3 V and very high current densities (in the μA range) to selected $Li@C_{60}$ molecules, 14 ion cage coordinations are unambiguously identified. To the best of our knowledge, this represents by far the largest number of accessible, reversible, and stable states observed for a surface-bound single molecule switch." to "By applying electric fields in excess of 3 V and very high current densities (in the μA range) to selected $Li@C_{60}$ molecules, 14 distinct ion cage coordinations are statistically populated and unambiguously identified. To the best of our knowledge, this represents by far the largest number of accessible, reversible, and stable states observed for a surface-bound single molecule switch. However, we recognise the current lack of final-state control compared to the degree of hierarchical control at low bias voltage reported for the rotation of Sc_3N inside C_{80} [7]."

- **With same goal, it is probably useful to cite work where superatom states are introduced to a broad audience, such as Acc. Chem. Res. 44, 360 (2011), since the concept is general but may not be very familiar.**

→ We have included the referee's suggested article, as well as a review article of our own that gives a background on SAMOs, to ensure that the reader can find the information they may require easily. The two added references are now **[33]** and **[34]**.

REVIEWER #3 (Prof Philip Moriarty, School of Physics and Astronomy, University of Nottingham)

Reviewer #3 recommends our paper for publication following minor changes and states "*I enjoyed this paper very much. It's a tour de force analysis of very high quality STM images and dI/dV spectra of Li@C60 on Au(111)*":

- **"The following work demonstrates for the first time 14-state switching behaviour of individual Li@C60 molecules condensed on a Au(111) surface using low temperature, ultrahigh-vacuum scanning tunnelling microscopy and spectroscopy." This is not at all the most compelling closing line for the introduction. It's not so surprising that this has been achieved "for the first time" given the narrowness of the system as described. The much more general result is that the authors have succeeded in exploring the potential energy landscape inside an endofullerene via careful and sophisticated analysis of variations in SAMO orbitals.**
  - → We thank the referee for his insights. We take this opportunity to improve our introduction following his recommendations. We have made the changes below:
  - → "for the first time" has been removed from the last sentence of the introduction: "The following work demonstrates a 14-state switching behaviour of individual Li@C_{60} molecules condensed on a Au(111) surface using low temperature, ultrahigh-vacuum scanning tunnelling microscopy and spectroscopy."
  - → The following concluding sentence was added: "Furthermore, careful analysis of variations in the superatom molecular orbital energies (SAMO, [**33, 34**]) allows the potential energy landscape inside an endofullerene to be explored."

- **In Fig.2 (and other figures in the paper), the STM bias voltage is +2.5 V and yet the key SAMO resonance is at 2.1 V. The obvious question to ask is why "spectroscopic imaging" wasn't carried out at 2.1 V so as maximise the contribution of that SAMO state to the images? A constant height dI/dV map acquired at 2.1 V would be very interesting to see. Do the authors have these data, or could they quickly acquire an image of this type to add to the paper?**
  - → The reason for performing topographic imaging at 2.5 V is to ensure our tunnelling signal integrates over the entire S-SAMO resonance. Hence the electronic contrast in STM images will be dominated by the S-SAMO over Li@C_{60} as opposed to over C_{60}. This is the main reason why the presence of Li in the cages can be so easily identifiable in measurements such as those reported in Fig. 2, Fig. S1, and Fig. S5. See also our previous work (reference [35]) and that of Yamada *et al*. (reference [26]).
  - → We have indeed performed dI/dV imaging at energies related to the S-SAMO resonance, and also at all other energies, including the other SAMO resonances for both Li@C_{60} and C_{60}. For C_{60}, these dI/dV images are already published in [35], see Fig. S10 of the S.I. We disagree with the referee that adding a single dI/dV image

acquired at about 2.1 eV would benefit the reader. The usefulness comes only about when reporting several dI/dV images acquired over the full range of SAMO + LUMO energies and providing the reader with a proper comparison between these (and with C_{60}). This is well beyond the scope of the present paper. However, we report here the two requested dI/dV images acquired on the S-SAMO of C_{60} (3.94 V) and Li@C_{60} (2.12 V) for comparison. Both S-SAMOs look fairly identical (white blob). Experimental conditions: 320 Hz modulation with 20 mVpp.

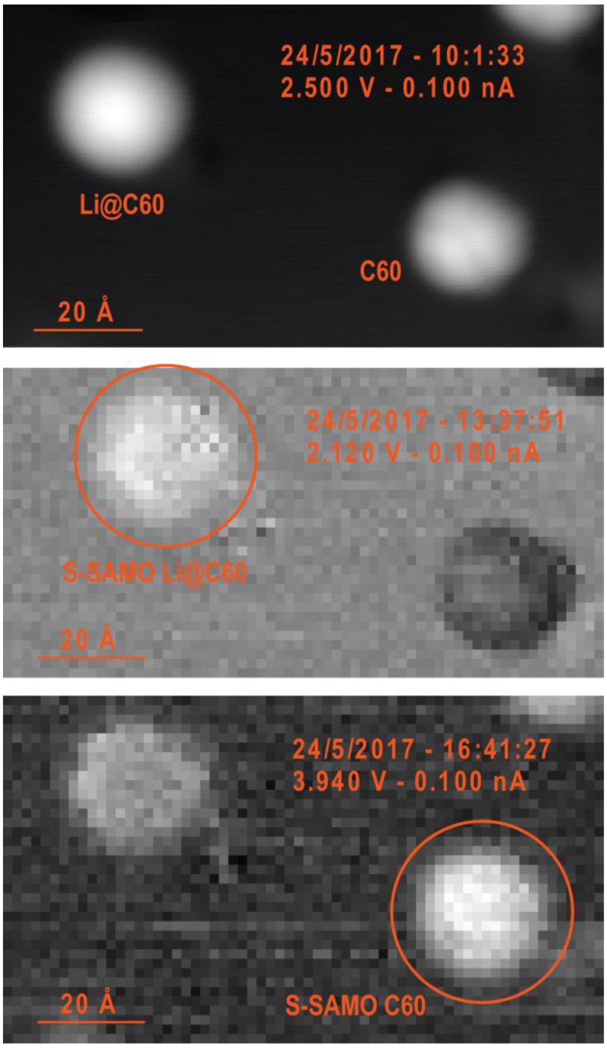

Figure: The top image is an STM image acquired at 2.5 V over two isolated fullerenes. The left one is a Li@C_{60} molecule, whereas the right one is a C_{60} molecule. This can easily be distinguished from their LDOS as acquired by single-point constant-current dI/dV spectroscopy.

The two images below are dI/dV images acquired at energies corresponding to the S-SAMO of each molecule.

The middle dI/dV image is acquired at 2.12 V corresponding to the S-SAMO resonance of the Li@C_{60} molecule. The bottom dI/dV image is acquired at 3.94 V corresponding to the S-SAMO resonance of the C_{60} molecule.

- **What's intriguing is that the Li never seems to reach a global minimum? Am I correct in saying this or do I misunderstand the authors' conclusions? Do the authors have any evidence (from, e.g. a combination of DFT and NEB barrier calculations) for the depth of the various potential minima in the cage?**

→ The referee is only partly correct. There exists an energetically favourable isomer (global minimum): the native state with the Li atom located furthest from the surface), i.e. level 1. However, our statistical analysis reveals that all other isomers (Levels 2, 3/4, 5 and 6) are equally probable.

→ We draw the referee's attention to references [16] and [25] that describe calculated barriers for Li movement between the various positions within the C_{60} cage. We discuss this on page 12: "*…we would anticipate an activation of 40–110 meV in line with the theoretically predicted energy barriers involved in the diffusion of the Li atom within a C_{60} cage [16, 25]*." We note that these calculations relate to gas phase molecules. These barriers will change (and we observe this by the reduction from 20 to 14 isomers) due to nearest-neighbour cages (intermolecular hybridisation) and the substrate (making Level 1 a global minimum).

- **The labelling of Fig.4(f) and Fig. 4(g) is the wrong way round.**

→ We thank the referee for noting this error. We have fixed this in the manuscript.

- **I think it's a "reach" to claim that Fig. 5 is direct evidence of Li ejection. There is no direct chemical information in the images. A highly localised (!) photoemission experiment could provide that direct information but the claim that Li has been ejected relies on the authors' interpretation of the data. It's certainly strong, credible evidence but I don't think it can be described as "direct".**

→ We agree with the referee that the STM images contain no chemical information. The chemical information lies in the comparison of the c.c. dI/dV spectra before and after excitation, which clearly (perhaps not directly) reveals that the result of the excitation is to transform a Li@C_{60} molecule into a C_{60} one.

→ Changes to manuscript: We have removed the word "direct" to our Li ejection description in the Caption of Fig. 5

- **Inelastic tunnelling spectroscopy could in principle be helpful in monitoring the vibrational modes of the Li. Have the authors attempted this measurement?**

→ We thank the referee for this suggestion. Besides being interesting on their own right, we don't think these measurements would enhance or strengthen any of the arguments we put forward in our paper. Hence we have not attempted these measurements. Here are our reasons for this:

  - We are aware of similar measurements on empty C_{60} cages [e.g.: K. J. Franke and J. I. Pascual, J. Phys.: Condens. Matter **24**, 394002 (2012)]. Consistent with these measurements, we naturally would expect a detailed Li@C_{60} vibrational structure to appear up to 200 meV in the d^2I/dV^2 spectrum. We note however that the excitations required for switching the Li position require an energy that is over an order of magnitude greater than this (hence our proposed mechanism of resonant tunnelling rather than direct IET).

MINOR ADDITIONAL CHANGES/EDITS TO MANUSCRIPT:

- Last line page 2, a "space" character too much is removed.
- References numbering changed throughout to accommodate for the introduction of refs [33] and [34] as suggested by referees.
- Page 4, "herring-bone" changed to "herringbone".

- Page 4, last paragraph, “images” changed to “features” in the sentence: “These are the features that are attributed…”
- Page 7, specific reference to the M- orientation is removed since statistical analysis was extended to both M- and m- orientations: “…native Li@C_{60} in the C_6 orientation.”
- Page 7, “Pz” changed to “P_z” for consistency.
- Page 9, specific reference to the M- orientation is removed since statistical analysis was extended to both M- and m- orientations: “Starting from the native C_6 ion-cage coordination of…”.
- Page 9, last paragraph, the word “a” is added in “… SAMO energy shifts as a function of …”.
- Page 9, author name corrected: “Schulze et al”.
- Page 10, author name corrected: “Schulze and co-workers”.
- Page 10, reported voltage value corrected: “…below 3.25 eV…”.
- Page 10, “$P_{x\text{-}y}$” changed to “$P_{x,y}$” for consistency.
- Page 11, “(see **SI.5**) and [**42, 46**]” changed to “(see **SI.5** and [**42, 46**])”.
- Page 12, a missing space is added in front of a sentence.
- Reference [35] is now updated with year, volume and page number.

MINOR ADDITIONAL CHANGES/EDITS TO SI:

- References numbering changed throughout.
- 1st paragraph in SI.1, a sentence is improved: “The data acquired at –2.5 V allow identification of the fullerene faces…”.
- 1st paragraph in SI.2, the word “as” changed for “, hence”.
- Caption Fig. S2, “a) to k)” changed to “(a) to (k)”.

CHANGES/EDITS TO MANUSCRIPT TO COMPLY WITH NATURE COMMUNICATIONS EDITORIAL CHECKLIST:

- References removed from abstract. Hence references numbering changed throughout.
- References reformatted in Nature Communications style.
- Checklist verified to be complete.

REVIEWERS' COMMENTS:

Reviewer #1 (Remarks to the Author):

The authors presented convincing answer and alterations of the manuscript in response to the referee's comments. I recommend the publication as is.

Alexey Popov

Reviewer #2 (Remarks to the Author):

The authors have adequately responded to the referee's comments. I recommend publication.

Reviewer #3 (Remarks to the Author):

The authors have addressed each of the referees' comments comprehensively and have modified their manuscript accordingly. I recommend publication of the paper as is.
